# A Supramolecular Reversible and Anisotropic Conductive Adhesive for Flexible Electronics

**DOI:** 10.1002/advs.75746

**Published:** 2026-05-15

**Authors:** Tongtong Li, Yahui Zhao, Ying Zhang, Yuquan Li, Xuan Ye, Hongyun Qiu, Zhaorui Zhang, Jiang‐Fei Xu, Shaobo Ji

**Affiliations:** ^1^ State Key Laboratory of Bioinspired Interfacial Materials Science Institute of Functional Nano & Soft Materials (FUNSOM) Soochow University Suzhou China; ^2^ College of Nano Science and Technology (CNST) Soochow University Suzhou China; ^3^ Key Lab of Organic Optoelectronics & Molecular Engineering Department of Chemistry Tsinghua University Beijing China

**Keywords:** flexible electronics, reversible connections, stretchable adhesives, supramolecular nanocomposites

## Abstract

Stretchable sensors are widely used in wearable devices and flexible electronics for their unique advantages in flexibility. However, assembling and integrating stretchable devices face challenges due to the low deformation tolerance of soft‐rigid connections. Moreover, in manufacturing flexible electronics, convenient and reversible connections like soldering that enable on‐demand replacement of soft units remain unavailable. Here, we report a supramolecular conductive adhesive (SCA) based on a nanocomposite of thermoresponsive polymers and conductive fillers. SCA applies to diverse substrates such as plastics, rubbers, and metals with robust mechanical connections and stable anisotropic electrical conductivity. SCA‐connected stretchable systems exhibit excellent tensile tolerance, with Au@SEBS connected to rigid units achieving mechanical stretchability exceeding 300% strain and electrical stretchability over 80% strain. SCA is also directly applicable to traditional electronic components, with reusability resembling traditional solders. At 80°C, SCA can be reversibly detached, and without renewal, SCA maintains its original connection strength and electrical stretchability over 5 reuse cycles. The reversible connections enable on‐demand replacement of stretchable units in flexible electronics for device repair or customization. This reduces the cost of flexible electronics and significantly extends their overall service life, holding broad potential in the more sustainable production of flexible electronics.

## Graphical abstract

Supramolecular network‐based nanocomposites are developed as a stretchable conductive adhesive for flexible electronics. It's as convenient as traditional solders and constructs anisotropic conductive and robust stretchable connections among diverse soft/rigid materials. It is suitable for the integration of flexible/stretchable electronics and provides the opportunity to renew fragile soft parts in devices and on‐demand sensor replacement, endowing extended device lifespan.

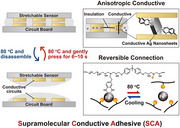

## 1 Introduction

Stretchable sensors are used in wearable devices and electronic skin, finding wide applications in health management and human‐machine interactions [1, 2, 3, 4, 5]. Their advantages primarily stem from their softness and flexibility, enabling them to conform to irregularly shaped substrates or biological surfaces and undergo deformation while retaining stable sensing interfaces [6, 7, 8]. Compared to bendable sensors, stretchable sensors accommodate a broader spectrum of deformations and deliver superior practical utility in wearable systems. However, soft materials in stretchable sensors differ from rigid materials in traditional electronics or flexible printed circuit boards (the Young's modulus of polyimide substrate is usually >1 GPa, thus also considered as “rigid” here) [9]. Mechanical property mismatch would cause stress‐strain concentration at soft‐rigid connections during deformation, while chemical incompatibility would result in weak soft‐rigid interfacial strength. These issues weakened the connections between soft stretchable sensors and rigid signal‐processing electronics [10], leading to their easy failure when deformed, thereby restricting the stretch tolerance and service life of integrated flexible devices [11, 12]. With advancements in flexible technology, increasingly high‐performance flexible and stretchable sensors have emerged [13, 14, 15, 16, 17, 18], while their convenient, stable, and low‐cost integration into flexible devices remained an urgent challenge [19, 20, 21, 22, 23, 24, 25].

**FIGURE 1:**
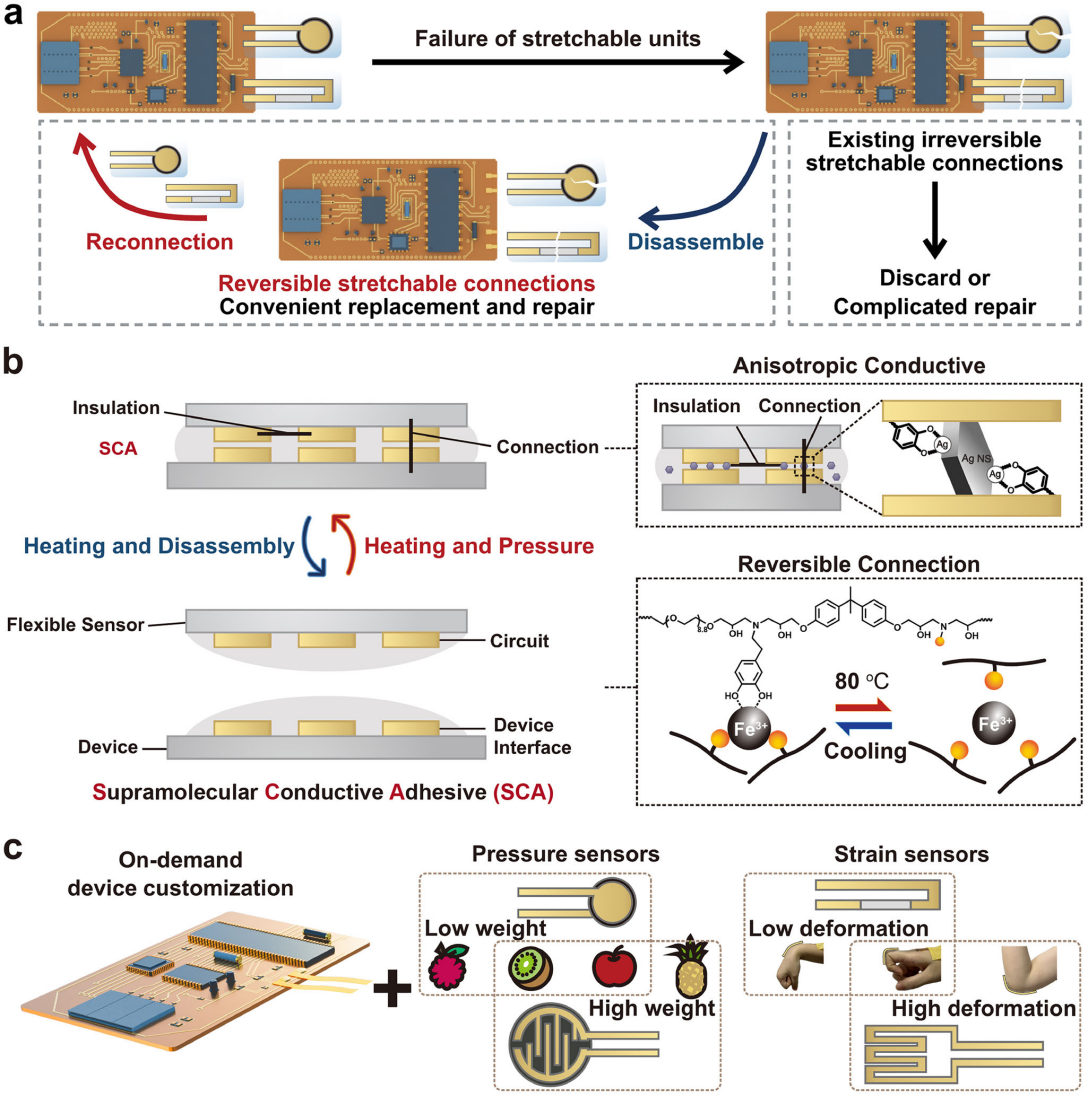
Interfacial connection by the supramolecular conductive adhesive (SCA). (a) Development of reversible stretchable connections would enable easier repair of flexible electronics. (b) Anisotropic conductivity and reversible connection of the stretchable connection. (c) Integration and on‐demand replacement of stretchable sensors for flexible device customization.

Recent advances have yielded conductive adhesives and connection strategies for interfacial connections between soft and rigid materials. Notable approaches include: Au@SEBS‐based connections exploiting self‐adhesion of rubbers and biphasic interfaces [26]; stretchable anisotropic conductive films with distributed gold particles enabling adhesion via thermocompression [27]; covalent interfacial reactions establishing stable mechanical bonds and reliable electrical pathways across diverse materials [28]. However, reported soft‐rigid connection approaches were typically irreversible and required complex and laborious processes. These issues restricted their practical usability and, more critically, prevented flexible interconnects from achieving the on‐demand sensor and electronic component replacement, which is common and convenient with soldering techniques in rigid systems. On‐demand replacement of stretchable sensors and other flexible components could be essential for flexible electronics. First, soft units undergo more rapid degradation than rigid ones and circuit boards, where replacement of soft parts enables easier repair of the integrated electronics and extended lifetime (Figure 1a). Second, diverse application scenarios require distinct sensing units, and on‐demand sensor replacement could realize low‐cost device customization. These advantages would significantly enhance the usability and reduce the manufacturing costs of flexible electronic devices. Therefore, taking the existing solders as a benchmark, developing a universal stretchable conductive adhesive that achieves convenient, stable, and reversible soft‐rigid connections would provide a promising approach for assembling and integrating flexible devices.

Supramolecular adhesives have achieved stimulus‐responsive and reversible binding for diverse substrates such as metals and plastics [29, 30, 31, 32, 33, 34, 35]. Compared to traditional thermoplastic adhesives, noncovalent bonds in supramolecular adhesives dissociate under milder temperatures for controlled separation of bound units, thereby exhibiting gentler thermal response ranges that are more compatible with soft materials [36, 37, 38, 39]. Moreover, during repeated usages, supramolecular adhesives exhibit nearly no loss in binding strength, making them strong candidates as matrix materials for desired stretchable adhesives [40, 41, 42]. To impart conductivity, conductive fillers must be incorporated into adhesive matrices. Anisotropic conductive films simultaneously provide through‐thickness conductivity, in‐plane insulation, and interfacial connection capabilities, offering a design paradigm for supramolecular conductive adhesives [43, 44, 45, 46]. By controlling the conductive fillers, it's possible to construct vertical contact between fillers and substrates for conductivity while preventing lateral conductive networks via filler dispersion, thus achieving anisotropic conductivity that enhances the application flexibility of supramolecular conductive adhesives.

Guided by the above rationale, we fabricated a nanocomposite using supramolecular polymers and silver nanosheets to develop an anisotropic conductive, mild‐temperature‐responsive supramolecular conductive adhesive (SCA) (Figure 1b). SCA's matrix comprised a supramolecular adhesive (SA) based on Fe^3^^+^‐crosslinked catechol‐functionalized polymers, with silver nanosheets (AgNS) as conductive fillers. Coordination bonds between catechol groups and Fe^3^^+^ provided cohesion that conferred high interfacial binding strength; these noncovalent interactions dissociate at mild temperatures (80°C), enabling separation of connected interfaces; upon cooling, they could spontaneously reform to reestablish interfacial connections, thereby allowing SCA to achieve thermoresponsive reversible bonding. The low conductive filler content (10 wt%) ensured anisotropic conductivity while keeping high interfacial conductivity. SCA exhibited user‐friendliness comparable to conventional solders, forming robust connections within 6–10 s at 80°C. It applies to diverse substrates, including plastics, rubbers, and metals, achieving adhesion strengths of 1.07 MPa for PI (flexible PCB substrate), 1.32 MPa for Cu, and 1.77 MPa for chip‐Cu interfaces. SCA constructed stretchable connections between stretchable components and diverse substrates, enabling exceptional deformation tolerance: Au@SEBS‐PI interfaces withstand >300% mechanical strain and >80% electrical stretchability. SCA demonstrated excellent reusability and removability, facilitating on‐demand replacement of flexible and stretchable sensors (Figure 1c) without significant adhesion degradation after repeated usage. This universal and reversible SCA not only established convenient, robust, and reversible device connections with anisotropic conductivity but also enabled multiple reuses and on‐demand component replacement. It enabled easier repair and extended the service life of flexible electronics and provided a promising system for low‐cost and sustainable integration and customization of flexible devices.

## 2 Results and Discussion

### 2.1 Preparation and Optimization of Supramolecular Conductive Adhesives

**FIGURE 2:**
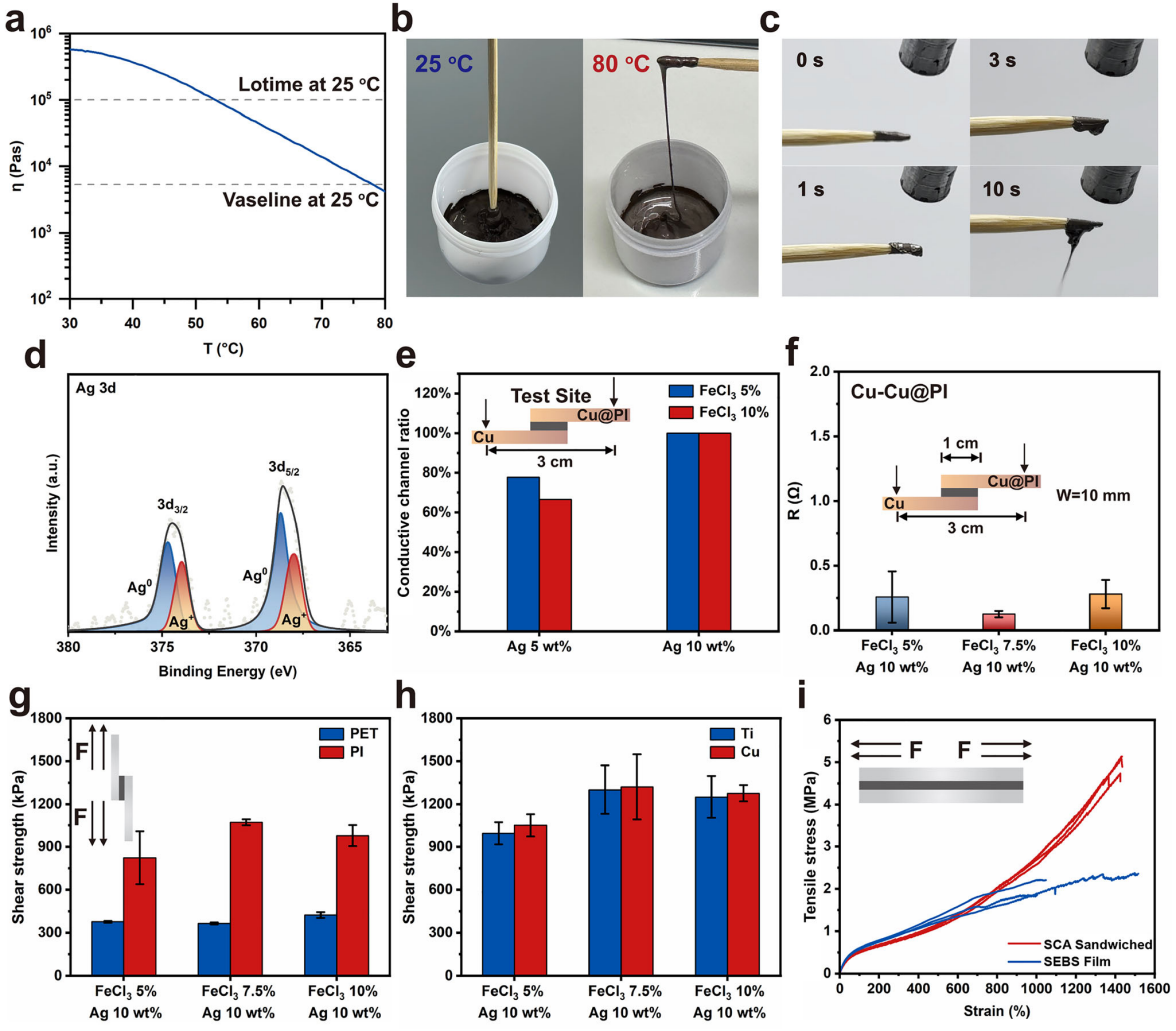
Formula optimization of SCA. (a) The viscosity (ɣ̇ = 0.1 s^−^^1^) of optimized SCA with 7.5% FeCl_3_ and 10 wt % of AgNS. Photos of the flow state of optimized SCA under (b) hot plate heating or (c) hot air gun heating. (d) XPS result of SCA. (e) Conductive channel ratio of SCA with different contents of AgNS. The total number of tested channels was 9. Bonding conditions: 80°C, 30 min, 5 kPa. (f) Resistance of Cu‐Cu@PI connected by SCA with different FeCl_3_ contents. Bonding conditions: 80°C, 30 min, 5 kPa. Lap shear tests of (g) plastics and (h) metals connected by SCA with different FeCl_3_ contents. Bonding conditions: 80°C, 30 min, 5 kPa. (i) Stretching results of optimized SCA sandwiched in SEBS films. Bonding conditions: 80°C, 6–10 s, 5 kPa.

According to previously reported methods [47], catechol‐functionalized polymer (CP) was synthesized by grafting dopamine (DOPA) onto epoxy resin chains (Figure S1). SA was yielded via crosslinking the synthesized CP with a defined quantity of FeCl_3_. And SCA was produced by further incorporating a small amount of AgNS. FT‐IR spectra of both SA and SCA (Figure S2) exhibit O−H stretching vibrations at 3270 cm^−^^1^ and 3200 cm^−^^1^, along with a bending vibration at 1184 cm^−^^1^, confirming abundant catechol groups and indicating coordination bond formation between Fe^3^^+^ and catechol moieties. For the optimally formulated SCA, when the temperature increased from 30°C to 80°C, its viscosity (at ɣ̇ = 0.1 s^−^^1^) decreased from 5 × 10^5^ Pa·s (comparable to asphalt) to 4×10^3^ Pa·s (comparable to Vaseline at rt) (Figure 2a). This confirmed that SCA could flow and detach from substrates at 80°C, guaranteeing its reversibility as a solder. SCA could respond to both direct heating (e.g., hot plate, soldering iron) and indirect methods (e.g., heat gun) in seconds, matching the convenience and feasibility of conventional solders (Figure 2b,c; Movie S1). Benefiting from the excellent temperature responsiveness of SCA, the requirement for bonding pressure was significantly reduced. Cross‐sectional SEM images reveal that under a bonding pressure of 5 kPa or manual finger pressure (estimated ca. 4–8 kPa), the silver nanosheets stack vertically, thereby enabling anisotropic conductivity (Figure S3). This relatively low‐temperature, rapid soldering process is beneficial for soft polymer‐based stretchable systems. SEM and EDS images of heat‐pressed SCA (Figure S4) revealed non‐contacting AgNS within planes, forming vertically aligned anisotropic conductive pathways as expected; Fe^3+^ was uniformly distributed, confirming that the supramolecular coordination network forms a homogeneous structure within the material system, which endows the material with favorable mechanical properties. The optimization of SCA was conducted by (1) varying the Fe^3^^+^ crosslinker ratio, which tunes its mechanical properties, interfacial adhesion strength, and temperature responsiveness; (2) adjusting AgNS content that determines its electrical characteristics.

Following reported protocols, the SA was first prepared with Fe^3^^+^‐to‐DOPA molar ratios of 5% (SA_5_), 10% (SA_10_), and 15% (SA_15_). Lap‐shear tests of flexible PET substrates bonded by SA_5_, SA_10_, and SA_15_ yielded shear strengths of 307 ± 36 kPa, 502 ± 77 kPa, and 468 ± 42 kPa, respectively (Figure S5a). Adhesion of SA initially rose, then declined with increasing Fe^3^^+^ content. The reason was that increasing the supramolecular crosslinker Fe^3^^+^ content strengthened the cohesion and adhesion of SA; while when the cohesion was too high, the flowability of SA was compromised, leading to poor contact with substrates and low adhesion. SA_15_ was already rigid with negligible flowability at 80°C (Figure S5b), thus not used for further experiments.

Rheological characterization revealed that both room‐temperature and 80°C viscosities (at ɣ̇ = 0.1 s^−^^1^) of SA increased with higher Fe^3^^+^ content (Figure S6). At 80°C, SA_5_ displayed a viscosity of 118 Pa·s (resembling syrup), and SA_10_ had a viscosity of 566 Pa·s (comparable to salad dressing). They both maintained sufficient flowability for reversible adhesion/detachment at 80°C. Tensile tests of SA_5_/SA_10_ sandwiched in stretchable SEBS demonstrated stress‐strain curves identical to those of pure SEBS films (Figure S7), indicating that the mechanical properties of flexible/stretchable units would not be influenced by the additional SA, which is of significance in keeping the softness of integrated components. Consequently, SA_5_ and SA_10_ were selected for subsequent electrical optimization studies.

To introduce electrical conductivity, small amounts of AgNS were incorporated into SA matrices, yielding SCA_5_ and SCA_10_ with varying AgNS content. High‐resolution Ag 3d XPS spectra of SCA (Figure 2d; AgNS was increased to 40 wt% to enhance signal intensity) revealed peaks at 374.69 eV and 368.69 eV corresponding to Ag^0^ 3d_3/2_ and 3d_5/2_ orbitals, respectively, while peaks at 373.96 eV and 367.96 eV indicated the existence of Ag^+^ species. This indicated potential coordination between catechol groups and Ag^+^. The FTIR spectrum of SCA (Figure S2b) exhibited O─H stretching vibration shifts from 3270 cm^−^^1^ to 3200 cm^−^^1^, further supporting the coordination between catechol groups and Ag^+^. Thus, the CP employed in this study not only coordinated with Fe^3^^+^ to form reversible crosslinked networks but also facilitated surface‐coordination‐driven dispersion of AgNS. This mechanism suppressed AgNS aggregation tendencies, ensuring their optimal compatibility and dispersion within SCA. When connecting Cu and CU@PI films, both SCA_5_ and SCA_10_ established conductive pathways with 10 wt% AgNS, achieving 100% conduction across 1‐mm‐wide Cu@PI circuit interfaces with 0.1 cm^2^ contact area while maintaining excellent conductivity (Figure 2e; Table S1). Conversely, SCA containing 5 wt% AgNS exhibited open‐circuit occurrences. Consequently, 10 wt% AgNS loading was selected for subsequent experiments.

The application of SCA and subsequent hot‐pressing produced conductive layers of merely 10–30 µm thick, yet at 80°C, a partial supramolecular network would still be elastic, causing thickness recovery during aging and electrical failure at the connections. After aging for two days, SCA_10_ interfaces partially failed electrically, whereas SCA_5_ maintained stable conductivity even after 10‐day aging (Table S2). Motivated by this observation, we introduced SCA_7.5_ with 7.5% Fe^3^^+^ and 10 wt% AgNS to further tune the material properties toward simultaneously high adhesion strength and superior electrical performance. SCA_7.5_ exhibited comparable connection resistance to SCA_5_ and SCA_10_ (Figure 2f). After 10‐day aging, SCA_7.5_ maintained 0.15 Ω resistance (Table S2), and it was still below 0.5 Ω even after 1‐year storage (Figure S8). Therefore, with AgNS fixed at 10 wt%, the mechanical connection properties of SCA_5_ and SCA_7.5_ were then compared to finally determine the optimal formulation.

Their temperature responsiveness and interfacial adhesion strength were characterized. The viscosities of SCA_5_ and SCA_7.5_ at 80°C (ɣ̇ = 0.1 s^−^^1^) were 4091 Pa·s and 4089 Pa·s, respectively (Figure S9), exhibiting flow properties like jam and chocolate spread. Their sufficient fluidity enabled rapid thermal responsiveness at mild temperatures (80°C). Compared to SA, the significantly elevated and converging viscosities of SCA_5_ and SCA_7.5_ originated primarily from the addition of solid AgNS. Figure 2g,h, and Figure S10 demonstrated robust binding of SCA_5_, SCA_7.5_, and SCA_10_ with plastics and metals. Specifically, SCA_7.5_ achieved 1.07 ± 0.02 MPa lap shear strength with PI and 1.32 ± 0.23 MPa with Cu, which were comparable to SCA_10_ and significantly higher than SCA_5_. Moreover, despite the solid AgNS, SCA_7.5_ still preserved the mechanical properties of stretchable materials (Figure 2i). Furthermore, in practical applications, a short pressing time (6‐10 s) still enabled stable mechanical and electrical connections similar to those in the optimization experiments (Figure S11a–c). Facing the usage environment of wearable devices, in which the temperature might reach 40°C, SCA could maintain 90.73% adhesion (Figure S11d–f). These results indicated the reliability of SCA. Through comprehensive consideration of interfacial adhesion, temperature responsiveness, and electrical performance, the optimized SCA composition was set as 7.5 mol% FeCl_3_ and 10 wt% AgNS.

### 2.2 Mechanical Connection

**FIGURE 3:**
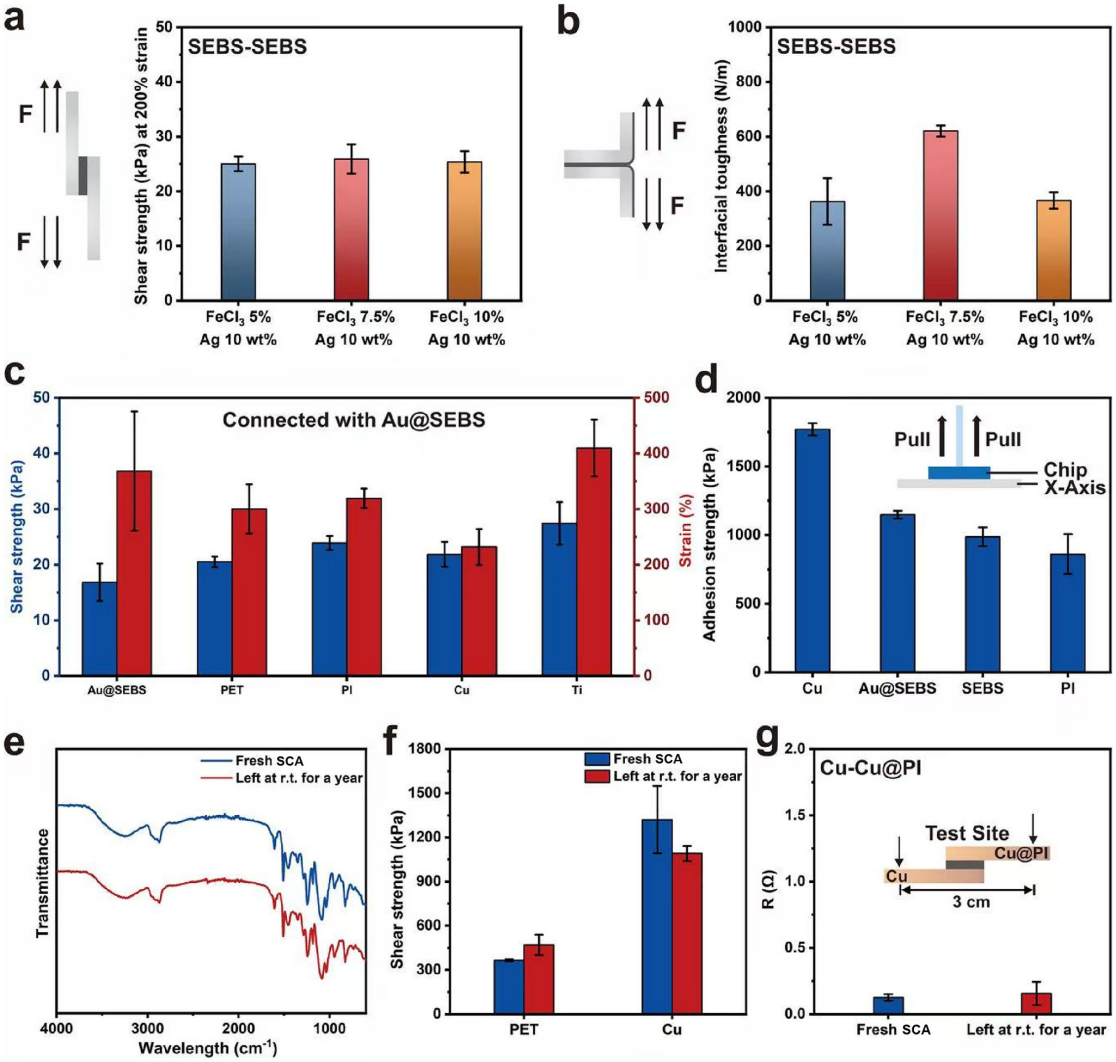
The mechanical connection by SCA. (a) Lap shear strength at 200% strain of SEBS films connected by SCA. Bonding conditions: 80°C, 6–10 s, 5 kPa. (b) 180° peel tests of SEBS‐SEBS connected by SCA. Bonding conditions: 80°C, 6–10 s, 5 kPa. (c) The shear strength and strain at break of SCA‐connected soft‐flexible/rigid combinations. Bonding conditions: 80°C, 6–10 s, 5 kPa. (d) Adhesion strength of SCA‐connected chips to the common materials used in flexible circuits. Bonding conditions: 80°C, 6–10 s, 5 kPa. (e) ATR FT‐IR spectra of fresh SCA and SCA left at room temperature for a year. (f) Lap shear tests of PET and Cu, and (g) connection resistance of Cu‐Cu@PI with fresh SCA and SCA left at room temperature for a year. Bonding conditions: 80°C, 30 min, 5 kPa.

To investigate SCA's adhesion strength in stretchable systems, mechanical tests of SCA bonded to SEBS rubbers and various substrates were performed. SCA‐connected SEBS films could tolerate over 400% tensile strain regardless of FeCl_3_ content, with similar lap shear strength at 200% strain (Figure 3a; Figure S12); while in the 180° peel test, SCA_7.5_ demonstrated the highest interfacial strength (Figure 3b; Figure S13). These results not only indicated that SCA could connect stretchable units but also confirmed that SCA_7.5_ had the best adhesion performance. Lap shear tests were then conducted on a stretchable conductor, Au@SEBS, with diverse substrates, for which the peel tests were not applicable due to the weak Au‐SEBS interface (Figure S14). Figure 3c and Figure S15 demonstrated the robust connection by SCA across stretchable, bendable, and conventional metallic materials. As common materials in flexible and rigid PCB, PI/PET‐Au@SEBS connections could withstand more than 300% strain, and Cu‐Au@SEBS connections tolerated over 200% strain, indicating the stable connection between stretchable units and flexible/rigid PCB by SCA. While for biocompatible Ti, which is widely used in implantable systems, Au@SEBS‐Ti connections sustained 410% average strain. For conventional electronic components, the adhesion strength of the Apple A8X CPU (17 mm × 16 mm) on various substrates was tested as an example. As shown in Figure 3d, SCA achieved adhesion strengths of 1.77 ± 0.04 MPa for metallic material Cu‐chip, 0.86 ± 0.14 MPa for bendable substrate PI‐chip, 0.99 ± 0.07 MPa for stretchable substrate SEBS‐chip, and 1.15 ± 0.03 MPa for stretchable conductive substrate Au@SEBS‐chip, respectively. This demonstrated that SCA could achieve robust adhesion with electronic components, thus enabling SCA to function as traditional solders in the integration of electronic devices and rigid/flexible circuits. Notably, weaker interfaces (Au‐SEBS, chip‐vertical holder, substrate‐instrument) always failed before SCA‐connected chip‐substrate interfaces during testing (Figure S16). For example, Au‐SEBS interfacial failure occurred in Au@SEBS‐chip tests, indicating that actual SCA‐chip adhesion strength exceeded the measured values.

Stability is a critical indicator for interfacial electrical interconnection. Environmental stress tests were conducted based on the application scenarios of SCA, such as high humidity and temperature during sports or in the summer. After aging at 45°C and 85% RH for 240 h, the adhesion strength did not degrade, demonstrating that SCA possessed good mechanical stability under damp‐heat conditions (Figure S17a,b). Moreover, PET‐SCA‐PET sandwich structures were immersed in deionized water at room temperature, 40°C, and 60°C, respectively, and no delamination or swelling was observed after immersion (Figure S17c). The results validated the reliability of SCA in the potential application environments.

As an adhesive targeting practical integration of flexible devices, the shelf life of SCA was investigated. The FTIR spectra were identical between fresh SCA and SCA stored under ambient conditions for more than a year (Figure 3e). And SCA retained excellent mechanical and electrical properties after 1‐year storage (Figure 3f,g). These results indicated that catechol groups within SCA resisted oxidation for at least one year, benefiting from the acidic Fe^3+^ ions, confirming SCA's sufficient stability. The above results confirmed that SCA could robustly connect diverse substrates, including metals, plastics, and rubbers, across soft‐soft, soft‐rigid, and rigid‐rigid interfaces. The soft‐rigid connections with SEBS‐based units exhibited high stretchability and demonstrated application potential for device integration. Simultaneously, SCA had convenient application protocols, simple storage conditions, and sufficient shelf life. These characteristics endowed SCA with compatibility with conventional electronic processing and integration systems.

### 2.3 Anisotropic Electrical Connection

**FIGURE 4:**
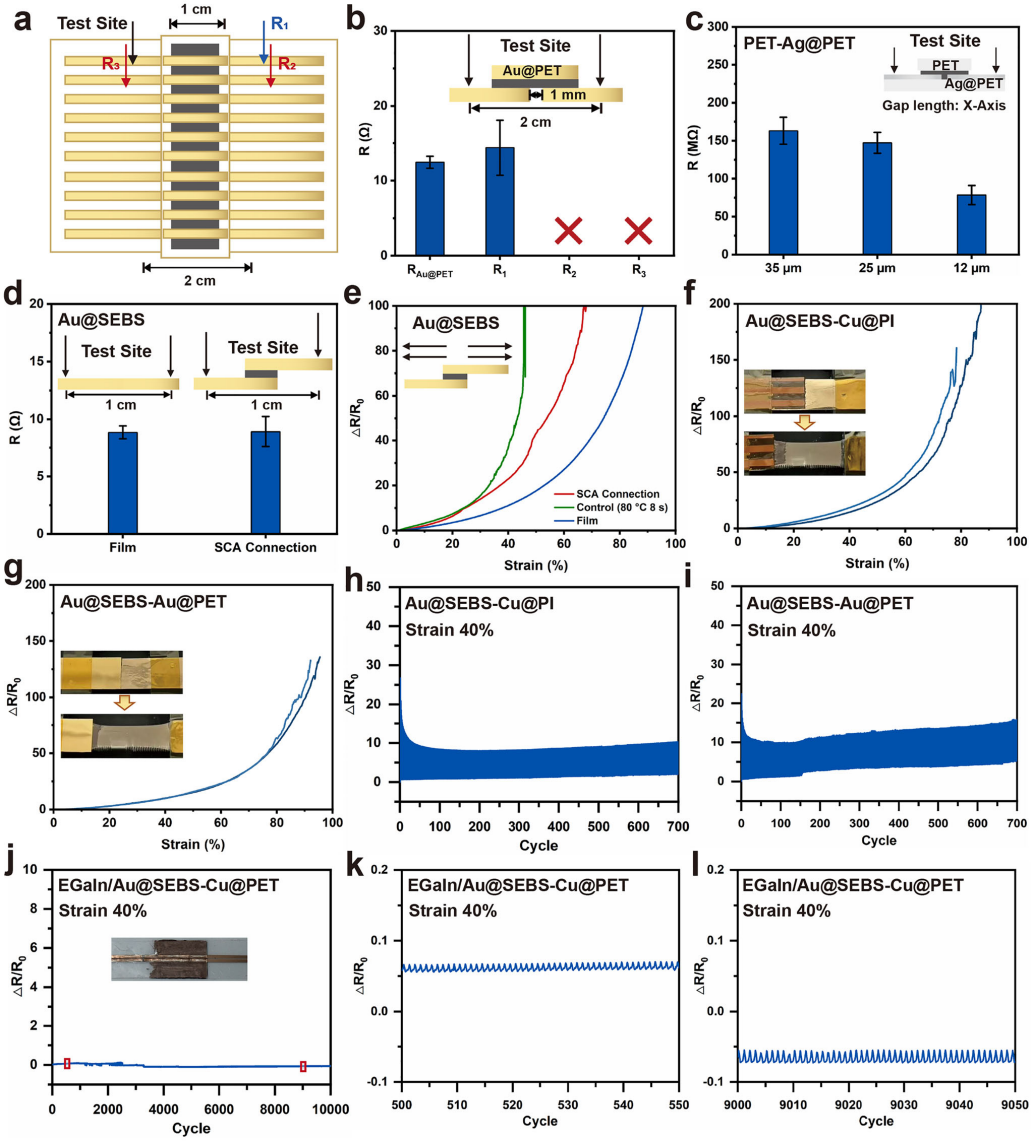
The anisotropic and stretchable electrical connection of SCA. (a) Schematics of anisotropic conductivity test. (b) Results of anisotropic conductivity tests. Bonding conditions: 80°C, 6–10 s, 5 kPa. (c) In‐plane insulation resistance under different spacings. The thickness of Ag is 100 nm. Bonding conditions: 80°C, 6–10 s, 5 kPa. d) Comparison of the electrical conductivity of plain Au@SEBS films or SCA‐connected Au@SEBS films. Bonding conditions: 80°C, 6–10 s, 5 kPa. (e) Comparison of the electrical stretchability of connected Au@SEBS films by self‐adhesion or SCA, and plain Au@SEBS film. Bonding conditions: 80°C, 6–10 s, 5 kPa. The electrical stretchability of SCA‐connected Au@SEBS to (f) Cu@PI, and (g) Au@PET. Bonding conditions: 80°C, 6–10 s, 5 kPa. (h) The electrical stability of SCA‐connected Au@SEBS‐Cu@PI. Bonding conditions: 80°C, 6–10 s, 5 kPa. (i) The electrical stability of SCA‐connected Au@SEBS‐Au@PET. Bonding conditions: 80°C, 6–10 s, 5 kPa. The resistance fluctuation was caused by the deformation of Au@SEBS during stretching. (j) The electrical stability of SCA‐connected EGaIn/Au@SEBS‐Cu@PET. Bonding conditions: 80°C, 6–10 s, 5 kPa. (k) Magnified view of the initial stage (cycles 500–550) and (l) magnified view of the later stage (cycles 9000–9050) from (j).

As flexible devices progressively miniaturize, existing connection methods increasingly struggle to achieve convenient and precise electrical connections. Anisotropic conductivity is a good solution to this challenge. The anisotropic conductivity of SCA was tested using Au@PET with patterned stripes of 2 mm widths and intervals (Figure 4a, top view; b insert, side view). The central section was fully covered by SCA to provide mechanical and electrical connections. The vertical interfaces exhibit conductivity comparable to Au@PET films, while adjacent strips remain non‐conductive (Figure 4b; Table S3). This confirmed the excellent anisotropic conductivity of SCA.

To further illustrate the dimensional limitation of the anisotropic conductivity, Ag@PET electrodes were fabricated with gaps of 12 µm, 25 µm, and 35 µm (Figure S18). SCA was applied onto the electrodes and pressed with PET sheets at 80°C for 6–10 s under 5 kPa. The results showed that the resistance between the electrodes decreased with decreasing gap; however, even at the narrowest gap of 12 µm under 20 V voltage, the in‐plane resistance remained as high as 78.49 ± 12.63 MΩ, indicating that the material can achieve in‐plane insulation at the micrometer scale (Figure 4c). We further fabricated Ag@PET stripes with a contact width of 400 µm and compared the resistance of the bare Ag@PET with that after the above bonding process. The two values were comparable, suggesting good interfacial contact under the current bonding conditions, with the interface resistance contributing negligibly to the total resistance (Figure S19).

The environmental stability of the electrical connection was also confirmed. As shown in Figure S20a, after aging at 45°C and 85% RH for 240 h, the interface resistance remained almost unchanged, demonstrating that SCA possessed good electrical stability under damp‐heat conditions. Moreover, after 48 h of continuous immersion and upon heating to 60°C, the LED‐Au@SEBS flexible circuit still emitted light normally with no obvious attenuation in luminous intensity, indicating that SCA could maintain good electrical connections facing water and elevated temperature conditions (Figure S20b and Movie S2). SCA also maintained excellent anisotropic conductivity after aging (Figure S21 and Movie S3). In thermal cycling tests, the interface was cycled between 20°C and 60°C. Throughout the cycling process, the resistance fluctuation remained below 80%, and the resistance returned to near its initial value after cycling (Figure S22). In current stress tests, SCA‐bonded Cu@PI interfaces exhibited stable resistance under both gradient current loading from 0.1 A to 0.5 A and continuous current loading at 0.5 A for 1 h, with no performance drift or failure induced by Joule heating (Figure S23). Collectively, these environmental stress tests validated the reliability of SCA.

For stretchable connections, SCA was pressed at 80°C for 6–10 s to connect stretchable conductive Au@SEBS for characterizing conductivity, electrical stretchability, and cyclic durability. As shown in Figure 4d and Table S4, SCA‐connected Au@SEBS exhibited conductivity equivalent to plain Au@SEBS films, confirming negligible interfacial resistance from SCA. Under the same conditions, adhesive‐free Au@SEBS‐Au@SEBS encountered electrical failure (ΔR/R_0_> 100) at ∼45% strain, whereas SCA‐connected Au@SEBS‐Au@SEBS could withstand ∼70% strain (Figure 4e). SCA connected soft‐rigid combinations maintained ΔR/R_0_ = 20 when Au@SEBS‐Cu@PI were stretched to 40% strain and Au@SEBS‐Au@PET to 50% strain, while achieving >80% electrical stretchability for Au@SEBS‐Cu@PI and >90% for Au@SEBS‐Au@PET (Figure 4f,g). This electrical stretchability was sufficient for connecting various existing stretchable/flexible sensors. Au@SEBS‐Cu@PI and Au@SEBS‐Au@PET connected by SCA could withstand >700 cycles at 40% strain while maintaining electrical stability, with ΔR/R_0_ fluctuations originating mainly from resistance changes of Au@SEBS (Figure 4h,i). Though the cyclic performance of SCA was not satisfying, the reusability of SCA‐connected interfaces (see next section), coupled with replaceable soft components, enabled substantial practical service life. Due to the inevitable accumulated cracking of the evaporated metal layer in Au@SEBS during cyclic stretching, the conductor restricted the cyclic stretchability of SCA (Figure S24). To circumvent this failure mechanism, a thin layer of liquid metal was introduced to enhance the conductor's cyclic stretchability. As shown in Figure 4j‐l, the SCA‐bonded interface of EGaIn/Au@SEBS‐Cu@PET stripes with a width of 500 µm could withstand 10 000 cycles at 40% strain, with no significant resistance variation throughout the cyclic stretching process. After cyclic testing, the interface maintained excellent anisotropic conductivity (Figure S25). Both soft‐soft and soft‐rigid interfaces connected by SCA had excellent conductivity, good electrical stretchability, and stable electrical performances (Table 1). SCA achieved universal, stable, long‐term anisotropic conductive connections, demonstrating its potential for stretchable and flexible electronic device integration.

**TABLE 1:** Comparison of flexible device integration methods.

Bondable materials	joint thickness	Mechanical tolerance	Contact resistance	Process pressure	Process temperature	reusability cycles	contact resistance measurement methods	Ref.
ICA	30±5 µm	No data	8 × 10^−5^ Ω cm	No data	∼50°C in air, 15 min	No data	ASTM F1896‐98	WBICAs [48]
ACF	>50 µm	200 cycles@1 mm bending radius	<1Ω	1–5 MPa	>200°C	No data	No data	ACF [49]
ACF	13–250 µm	2000 cycles of 15% stretching (Au@PI‐EGaIn@PDMS), 2000 cycles of 50% stretching (EGaIn@PDMS‐EGaIn@PDMS), >70% stretching (EGaIn@PDMS)	0.76 Ω mm^−2^	<0.1 MPa	∼80°C, 10min	No data	Four‐point probe measurement method	S‐ACF [27]
ACA	>500 nm	10000 cycles@2 cm bending radius (Au@PET)	9 ohm	0.1 MPa	RT in air	No data	Probe station and precision source/measure unit	ACA [50]
Containing liquid metal	No data	>1000 cycles of Bending (high Ag E‐CASE on a PET substrate), 400% Stretching (E‐CASE integrated LED)	0.06–0.75 Ω cm^−1^	No data	∼100°C, 30min	No data	Four‐probe setup	E‐CASE [49]
low‐temperature metallurgical solders	0 µm	No data	No data	non‐pressure process	100°C, 10min	No data	No data	Sn‐Bi‐in‐xGa [51]
SEBS‐adherable material	0 µm	600 cycles of 50% stretching (Au@SEBS‐Au@SEBS, Au@SEBS/PET‐Au@SEBS), 180% stretching (Au@SEBS‐Au@SEBS), 200% stretching (Au@SEBS/PET‐Au@SEBS)	10 Ω sq^−1^	No data	RT in air	No data	square‐sized sample (0.5cm^2^)	BIND [26]
ACA	10‐30 µm	>700 cycles of 40% stretching (Cu@PI‐Au@SEBS, Au@PET‐Au@SEBS), >10 000 cycles of 40% stretching (Cu@PET‐EGaIn/Au@SEBS), 110% stretching (LED‐EGaIn/Au@SEBS)	<0.17 Ω cm^−2^	∼5 kPa	∼80°C in air, 6–8 s	7 cycles (LED‐Au@PET), 4 cycles (Au@SEBS‐Cu@PI)	Keithley DMM6500, Keithley 2450	SCA This work

### 2.4 Application and Cyclic Reusability

**FIGURE 5:**
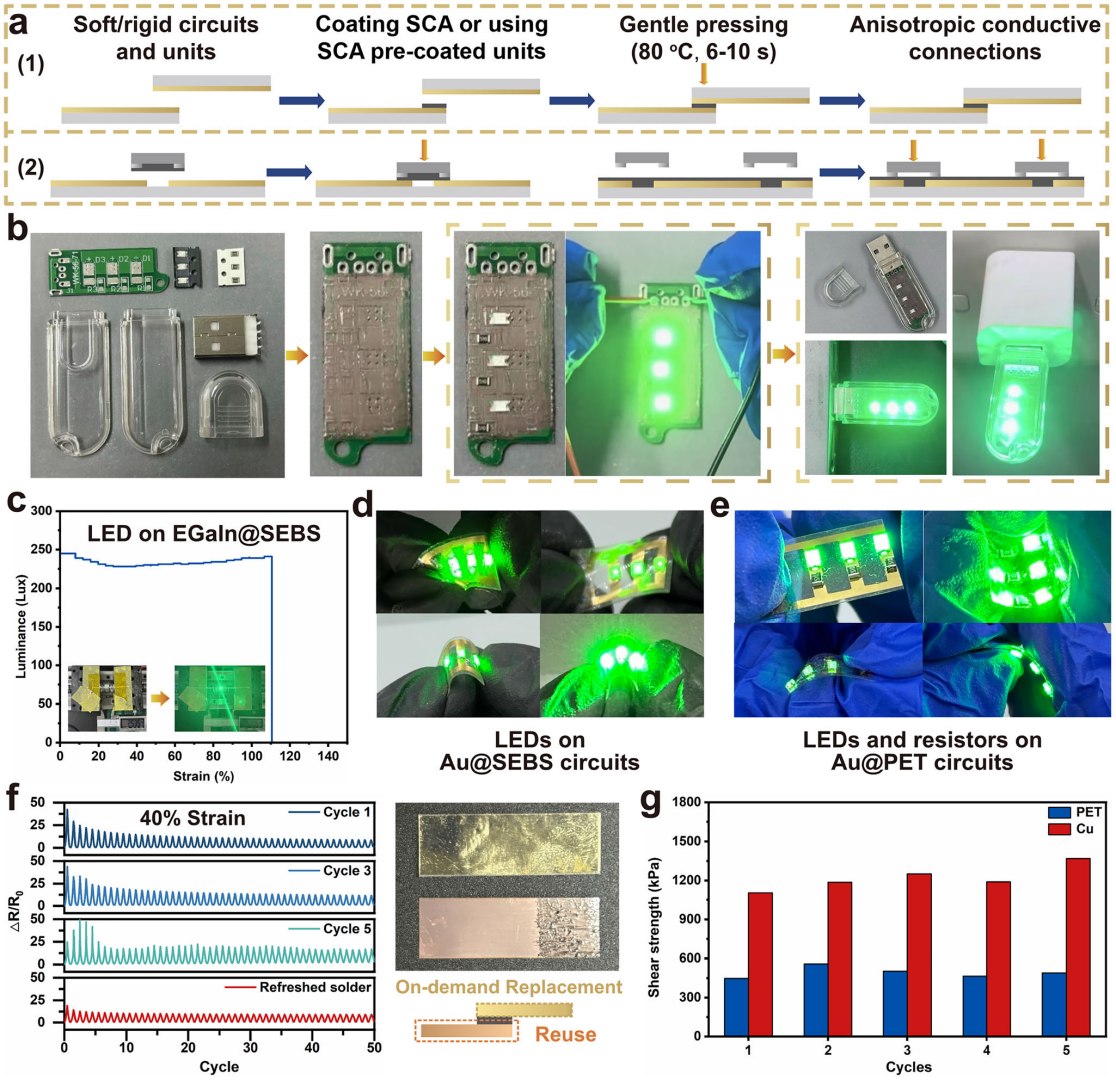
Rigid, flexible, and stretchable circuits construction and reusability of SCA. (a) Two connection scenarios using SCA. (b) Integration of a PCB, resistors (type 0805, 51 Ω), and LEDs (type 1206, green) by pre‐coating SCA on the PCB. Bonding conditions: 80°C, 6–10 s, manual finger pressure (estimated ca. 4–8 kPa). (c) The luminance variation of the SCA‐mounted LED on EGaIn@SEBS during stretching. Bonding conditions: 80°C, 6–10 s, manual finger pressure (estimated ca. 4–8 kPa). d) The SCA‐fixed LED on Au@SEBS circuits could withstand multiple deformations. Bonding conditions: 80°C, 6–10 s, manual finger pressure (estimated ca. 4–8 kPa). (e) The SCA‐mounted LEDs and resistors on Au@PET circuits could endure bending. Bonding conditions: 80°C, 6–10 s, manual finger pressure (estimated ca. 4–8 kPa). (f) The electrical stretchability of the SCA‐connected Au@SEBS‐Cu@PI after each reuse without adding new adhesive. The initial decrease in resistance arose from the property of Au@SEBS. Bonding conditions: 80°C, 6–10 s, 5 kPa. (g) Lap shear strength of SCA‐connected PET or Cu after each reuse. Bonding conditions: 80°C, 6–10 s, 5 kPa.

SCA demonstrated operational convenience and application flexibility comparable to conventional solders. Figure 5a illustrates two scenarios: (1) establishing connections by SCA via gentle pressing under suitable temperatures; (2) fixing components on circuits by pre‐coating SCA onto electronic components or circuit boards, followed by pressing under suitable temperatures (Movies S4 and S5). Owing to its anisotropic conductivity, SCA does not require precise coating at the electrode areas, imparting convenience of usage. To confirm this, SCA was used to integrate a simple circuit on a PCB (Figure 5b). The PCB was fully coated with SCA, and resistor blocks (0805 package, 0.40 mm × 1.25 mm contacts) and LEDs (1206 package, 0.70 mm × 1.30 mm contacts) were positioned and pre‐fixed by the tacky SCA. The board was then transferred to an 80°C hot plate with gentle pressing for 6–10 s to produce the USB‐interfaced LED arrays. This process was more straightforward than conventional soldering by lowering temperature requirements and avoiding short‐circuit risks due to anisotropic conduction. SCA could alternatively be pre‐coated on resistor/LED surfaces to achieve identical connections, through which simple flexible circuits were fabricated as demonstrations. LEDs and resistors were fixed on stretchable EGaIn@SEBS stripes, Au@SEBS circuits, and flexible Au@PET circuits. The deformation tolerance mainly depended on the performance of the circuit materials rather than the SCA connections: LEDs on EGaIn@SEBS stripes maintained stable illumination at 110% strain (Figure 5c); LEDs fixed on Au@SEBS circuits could withstand multi‐directional bending, stretching, and twisting (Figure 5d); LEDs/resistors mounted on Au@PET circuits endured omnidirectional bending (Figure 5e). These results validated the broad applicability of SCA across diverse systems.

The reusability of SCA is important for flexible devices, which would reduce production costs and extend overall device lifespan through on‐demand replacement of soft components. To investigate the reusability of SCA, it was applied to repeatedly connect diverse devices and materials. SCA constructed interfaces were disassembled at 80°C and reconnected with gentle pressing. The SCA‐connected LEDs on stretchable Au@SEBS circuits were easily replaced without damaging the soft substrate or recycled and reused (Figure S26a), exhibiting convenience resembling solders in traditional electronics and PCBs. Without additional SCA, different LEDs were successively replaced on the same site of Au@PET over 7 times while maintaining stable electrical connections (Figure S26b), demonstrating the good reusability and stability of SCA. In the SCA‐connected stretchable system Au@SEBS‐Cu@PI, the stretchable Au@SEBS could be replaced consecutively 4 times without additional adhesive while maintaining stable electrical stretchability (Figure 5f; Figure S27a). Noticeable performance degradation occurred during the fifth and sixth reuse cycles. The major reason was the adherence of Au on SCA due to the relatively weaker Au‐SEBS interfaces in stretchable units, which would interrupt the integrity of the SCA layer with an elevated amount (Figure S27b). However, SCA could be rapidly and completely removed from the surfaces using deionized water (Figure S27c), providing the opportunity to refresh the SCA at the connection points to reconstruct robust connections. The lap‐shear strength of SCA‐connected PET or Cu after cyclic usage indicated that the mechanical connections could be well maintained; the reusability of SCA was mainly limited by the properties of soft units rather than the performance of SCA itself. For instance, in the bendable Cu@PI‐Cu@PI system, the interface resistance exhibited no significant change over 4 reuse cycles (Figure S28). The reversible disruption and reformation of supramolecular networks endowed SCA with exceptional reusability, enabling rapid and repeated reuse under mild temperatures and complete post‐application cleaning.

### 2.5 On‐Demand Replacement of Stretchable Sensors

**FIGURE 6:**
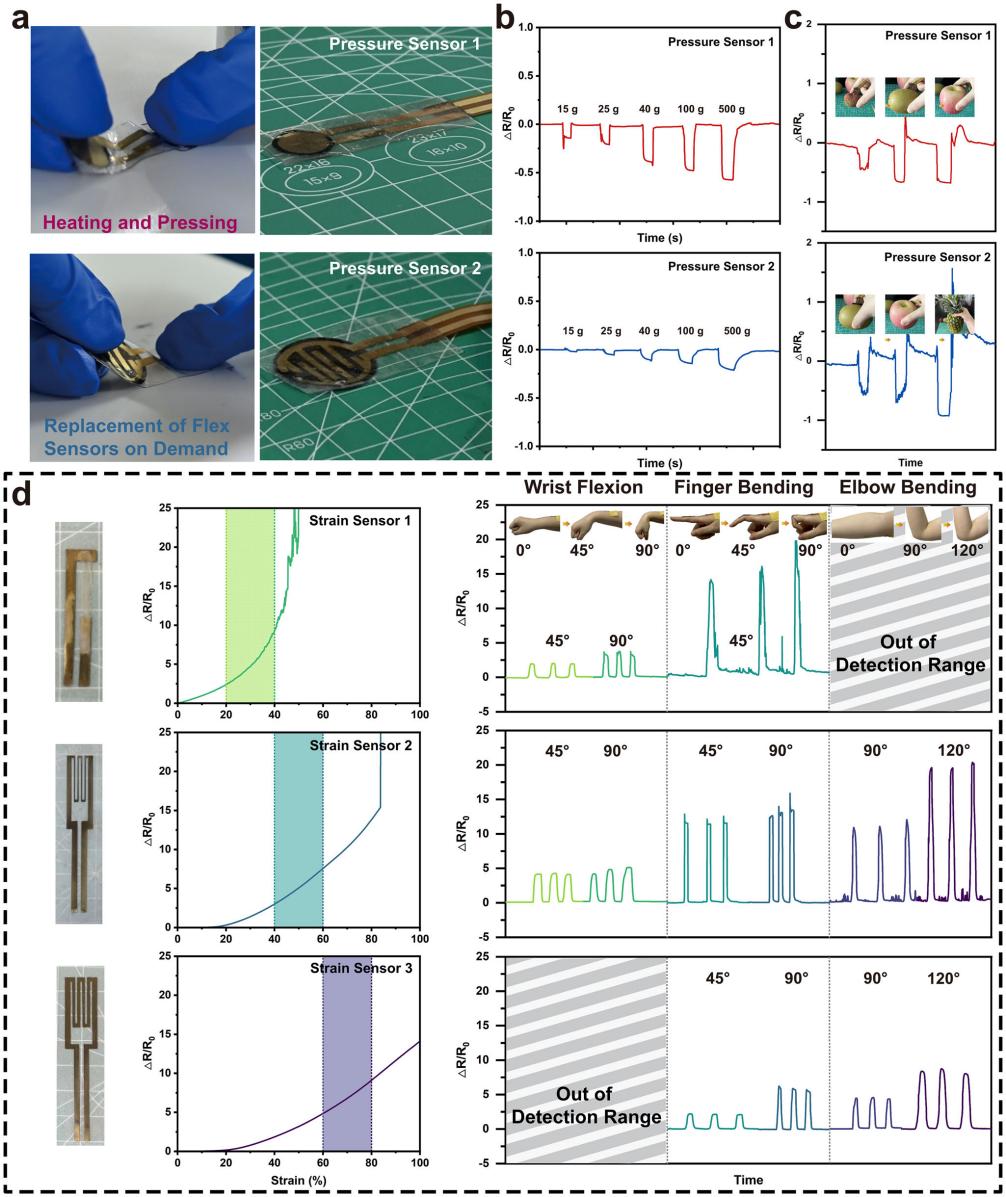
On‐demand replacement of stretchable sensors on a single connector with SCA for device customization. (a) Connection and replacement of different pressure sensors on a Cu@PI connector. Bonding conditions: 80°C, 6–10 s, manual finger pressure (estimated ca. 4–8 kPa). (b) The sensor performance of two pressure sensors. (c) Weight testing of fruits by replacing flexible pressure sensors on demand. Bonding conditions: 80°C, 6–10 s, manual finger pressure (estimated ca. 4–8 kPa). (d) Bending degrees of wrist, fingers, and elbow were tested by replacing stretchable strain sensors on demand. Bonding conditions: 80°C, 6–10 s, manual finger pressure (estimated ca. 4–8 kPa).

To demonstrate the application of SCA in on‐demand replacement of flexible sensors, a Cu@PI connector was employed to connect resistive strain/pressure sensors with electronic equipment. Stretchable sensors were connected by SCA on a hot plate with gentle pressing, and they could be replaced by other sensors under the same conditions (Figure 6a; Movie S6). The sensors had varied applicable ranges. Pressure sensor 1 was relatively sensitive in lower‐pressure regimes but could not distinguish high pressures, while pressure sensor 2 could distinguish high pressures but could not distinguish low pressures (Figure 6b). As a demonstration, SCA‐connected pressure sensors were used to detect object weights using fruits as test subjects. Fruits of different weights require distinct grasping forces. As shown in Figure 6c, pressure sensor 1 could measure lighter fruits like lychees, yet failed to distinguish between medium‐weight kiwifruits and apples; pressure sensor 2 output no signal when holding lychees, but distinguished apples from heavier pineapples. This on‐demand replacement of pressure sensors realized the customization of sensing devices according to the practical requirement and lowered the fabrication complexity and cost.

The device customization was further demonstrated by using different strain sensors to detect the bending of the finger, wrist, and elbow (Figure 6d; Figure S29). Stretchable resistive strain sensors were connected to sensing devices via the Cu@PI connector and SCA. The three strain sensors had different optimal sensing ranges, as marked in their sensing curves. Strain sensor 1 could detect low‐strain wrist bending with distinguishable angles, but its narrow detection range caused significant signal distortion when facing finger bending and completely failed for elbow motions. Strain sensor 3 suited medium/high‐strain finger/elbow bending with distinguishable angles, yet struggled to detect wrist flexion due to higher detection limits. Strain sensor 2 offered broad sensing coverage and exhibited stronger signals for high‐strain elbow bending, but could not distinguish bending angles for low/medium‐strain wrist/finger bending. Thus, the same electronic part could be used for different positions: strain sensor 1 for wrist; when detection on finger was needed, it could be replaced by strain sensor 3; and for elbow sensing, if stronger signals were required, the strain sensor could be further replaced by 2. This would save the effort of designing or fabricating redundant electronic circuits and devices. The reversible connection/reusability of SCA provided a stretchable substitution in flexible electronics for conventional solders and could reduce the operational expenditures and manufacturing costs to advance flexible electronics industrialization.

## 3 Conclusion

A universal reusable supramolecular conductive adhesive (SCA) has been designed and fabricated. Owing to the mild temperature‐responsive supramolecular networks, SCA enabled convenient, robust, and reversible connections between diverse materials, including plastics, rubbers, and metals, at 80°C with 6–10 s pressing. It applied to soft stretchable systems without compromising their mechanical properties. SCA possessed excellent anisotropic conductivity and could be used as conventional solders to integrate rigid electronic components and PCBs. When applied to stretchable systems, SCA established stable stretchable connections: Au@SEBS‐Cu@PI could withstand over 80% strain before electrical failure, while the value of Au@SEBS‐Au@PET exceeded 90%. They both sustained >700 stretching cycles at 40% strain with stable electrical performance. Though the cycle number was not satisfying, combined with its reusability, SCA could still extend the overall service life of stretchable devices in practical applications. SCA demonstrated exceptional reusability and removability, maintaining the connection strength through five reuse cycles while enabling convenient and rapid post‐application cleaning. Based on these properties, SCA realized convenient and adaptable replacement of stretchable sensors with varying measurement ranges and sensitivities on a single connector to electronic circuits, achieving on‐demand stretchable sensor replacement and device customization. SCA provided a universal integration solution bridging flexible/stretchable devices with traditional electronics. Its reversible properties enhanced manufacturing fault tolerance to reduce production costs, and its reusability facilitated the replacement of fragile soft units for easier repair. SCA unlocked new possibilities for flexible devices and could push forward more sustainable production of practical and customized flexible electronics.

## 4 Experimental Section

### 4.1 Materials

Diglycidyl ether of bisphenol A (DGEBA), poly (ethylene glycol) diglycidyl ether (EGDE, M_n_ = 500 g/mol), ferric chloride (FeCl_3_), and dopamine hydrochloride were purchased from Sigma‐Aldrich. Triethylamine (TEA) was purchased from Aladdin. Basic alumina was purchased from Innochem. Silver nanosheets (∼5 µm in diameter, BET: 0.80–1.45 m^2^/g) were purchased from XFNANO. Methanol was purchased from Beijing Tong Guang Fine Chemicals Company. The weights of the lychee, kiwi, apple, and pineapple are ∼25 g, ∼100 g, ∼200 g, and ∼1.5 kg, respectively. All reagents and solvents were used as received without further purification.

### 4.2 Synthesis of Catechol‐Functionalized Polymer (CP)

Dopamine hydrochloride (1.89 g, 10 mmol) was stirred with basic alumina (1.30 g) in methanol (30 mL) for 10 min and filtered. The filtrate was reacted with DGEBA (1.96 g, 5.0 mmol) and EGDE (2.50 g, 5.0 mmol) at 40°C for 48 h in a round‐bottom flask under N_2_ atmosphere and magnetic stirring to obtain CP. After the reaction, a yellow solution was obtained. ^1^HNMR were measured in CDCl_3_ solutions by a Bruker AVANCEIII HD‐400 NMR spectrometer at 298K.

### 4.3 Synthesis of Supramolecular Conductive Adhesive (SCA)

SCA was prepared by the following steps. The methanol solution of CP (6.0 mL, 2.0 mmol of DOPA unit) was mixed with TEA (0.368 g, 3.6 mmol) and FeCl_3_ (0.024 g, 0.15 mmol) under stirring. After 12 h at room temperature, it was sonicated for 1 h and dried under vacuum at 60°C for 4 h (to produce SA, the methanol would be fully removed with longer drying time). Then AgNS (0.708 g, 10 wt%) was mixed into the mixture by a planetary mixer (Rsimai Cyclone‐SR2000). It was then further dried under vacuum at 60°C for 2 h to completely remove methanol and TEA. The product SCA was not further purified. Air was not purposely avoided during the synthesis of SCA.

### 4.4 Characterization

Fourier transform attenuated total reflectance infrared spectra (ATR‐FTIR) were recorded by a Bruker V70 & Hyperion1000. X‐ray photoelectron spectroscopy (XPS) was recorded by an Axis Ultra DLD Kratos AXIS SUPRA. The dispersion of silver flakes after heating and pressing, along with the elemental distributions of Fe and Ag, was observed by scanning electron microscopy (SEM, ZEISS G500) with energy‐dispersive X‐ray spectroscopy (EDS). The viscosity was measured by a rotational rheometer (ThermoFisher Scientific, HAAKE MARS 40) at a shear rate ɣ̇ = 0.1 s^−^^1^. Thermal images were captured using a handheld thermal imager (FOTRIC).

### 4.5 Mechanical Characterization

The mechanical strength and lap‐shear strength were measured by a mechanical tester (YH‐9002) in tensile stretching mode. The stretching rates were all fixed at 60 mm/min. For a more precise comparison, the connected samples were pressed with 5 kPa pressure at 80°C for 6–10 s or 30 min. To demonstrate the convenience of the bonding process, manual finger pressing (estimated ca. 4–8 kPa, the value was calculated by pressing on a balance with a fixed contact area) was applied for certain samples under the same temperature and duration. The specific bonding conditions for each sample are indicated in the corresponding figure captions. The samples of sandwiched tensile experiments were cut into stripes with a width of 5 mm for tensile testing. The interfacial toughness of soft‐soft interfaces and soft‐rigid interfaces, and the connection strength between rigid materials, were measured by lap shear tests with a 10 mm × 10 mm overlapping area. The mechanical stretchability and electrical stretchability were measured in lap shear mode with a 10 mm × 10 mm overlapping area. The normal adhesion strength of the chip bonding was measured by a mechanical tester (INSTRON 34TM‐50) in tensile stretching mode. The bonding area was 16 mm × 17 mm, and the tensile rate was 60 mm/min.

### 4.6 Electrical Characterization

The electrical resistance was measured by a Keithley DMM6500 6.5‐digit multimeter, and the in‐plane insulation resistance was measured by a Keithley 2450 SourceMeter. Liquid metal lines were drawn on samples for better contact with the wires from the multimeter. To simultaneously obtain electrical and mechanical results, mechanical strain was applied by a mechanical tester. The stretching rate for electrical stretchability tests was 60 mm/min. The cycle stability of SCA‐connected Au@SEBS and commercial PI PCB or Au@PET was recorded after reaching a stable state (after about 50 cycles) due to the continuously decreasing resistance of Au@SEBS in the initial stretching and releasing cycles.

### 4.7 Fabrication of Stretchable Substrates, Conductors, and Sensors

SEBS (Tuftec H1221) films (thickness∼150 µm) were prepared by casting 20 mL of SEBS solution (13 wt% in toluene) into a glass mold (rectangles, length and width both 12 cm) and slowly removing the solvent by evaporation at rt. Metal deposition was done by electron beam evaporation with a Kurt J Lesker PVD75, at a rate of 0.5 Å/s for all samples. For Au@SEBS, a 45 nm thick Au layer was evaporated on the SEBS film. For Au@PET, a 70 nm thick Au layer was evaporated on PET substrates. For Ag@PET, a 100 nm thick Ag layer was evaporated on PET substrates. For Cu@PET, a 100 nm thick Cu layer was evaporated on PET substrates. EGaIn@SEBS was fabricated by rubbing liquid metal (75.5% Ga, 24.5% In) on SEBS with masks to form lines. EGaIn/Au@SEBS was fabricated by rubbing liquid metal (75.5% Ga, 24.5% In) on Au@SEBS with masks to form lines. The sensors were all based on SEBS film as a stretchable substrate with a 70 nm thick patterned Au layer evaporated on the SEBS substrate. The stress sensors consisted of Au@SEBS and piezoresistive materials. The strain sensors consisted of Au@SEBS and silver nanowires. The circuit patterns were realized by using masks with the desired patterns. The wearing experiments of sensors were approved by the Ethics Committee of Soochow University (ECSU, Approval No. SUDA20250609H08), and consent forms were signed by the participants.

### 4.8 Interface Connection and Disconnection Process with SCA (Reuse/Use Process)

For all the connections constructed in this work, they were realized through the following processes (except for the sample to compared mechanical properties): Heat the SCA to 80°C, apply it directly to the surface of the soft/rigid substrates, circuit boards, sensor interfaces, etc., and press the connection for 6–10 s, then it was cooled to r.t. for further usage or characterization. The thickness at the connection increased by 10–30 µm. To reuse the interface or disassemble the connections, heat the connection to 80°C, and tear off the interface. If a complete cleaning of the device interface was desired, it could be further washed with deionized water.

## Funding

This work is funded by the National Natural Science Foundation of China (52473218, 22525105, U24A20496); Basic Research Program of Jiangsu Province (BK20240826); Suzhou Key Laboratory of Surface and Interface of Intelligent Matter (Grant No. SZS2022011); the Gusu Innovation and Entrepreneurship Talent Program – Major Innovation Team (ZXD2023002).

## Conflicts of Interest

The authors declare no conflict of interest.

## Supporting information




**Supporting File 1**: advs75746‐sup‐0001‐SuppMat.pdf.

**Supporting file 2**: advs75746‐sup‐0002‐SuppMat‐MovieS1.mp4.

**Supporting File 3**: advs75746‐sup‐0003‐SuppMat‐MovieS2.mp4.

**Supporting File 4**: advs75746‐sup‐0004‐SuppMat‐MovieS3.mp4.

**Supporting File 5**: advs75746‐sup‐0005‐SuppMat‐MovieS4.mp4.

**Supporting File 6**: advs75746‐sup‐0006‐SuppMat‐MovieS5.mp4.

**Supporting File 7**: advs75746‐sup‐0007‐SuppMat‐MovieS6.mp4.

## Data Availability

The data that support the findings of this study are available from the corresponding author upon reasonable request.
