# Supplementary material for: A Supramolecular Reversible and Anisotropic Conductive Adhesive for Flexible Electronics
Source: Adv Sci (Weinh). 2026 May 15:e75746. Online ahead of print. doi: 10.1002/advs.75746 (PMC13335866; doi:10.1002/advs.75746)
Supplement: Supplementary file 1 — Supporting File 1: advs75746‐sup‐0001‐SuppMat.pdf. [file ADVS-9999-e75746-s007.pdf]

Supporting Information

A supramolecular reversible and anisotropic conductive adhesive for flexible electronics

*Tongtong Li, Yahui Zhao, Ying Zhang, Yuquan Li, Xuan Ye, Hongyun Qiu, Zhaorui Zhang, Jiang-Fei Xu, and Shaobo Ji**

Contents:

Supplementary Figures S1-S29.
Supplementary Tables S1-S4.
Supplementary Movies S1-S6:
Movie S1: The thermoresponsive behavior of SCA
Movie S2: Immersion test
Movie S3: Resistance measured by Keithley DMM6500 (6.5-digit)
Movie S4: An integrated circuit on SEBS
Movie S5: An integrated circuit on PET
Movie S6: On-demand replacement of stretchable sensors

Supplementary Figures

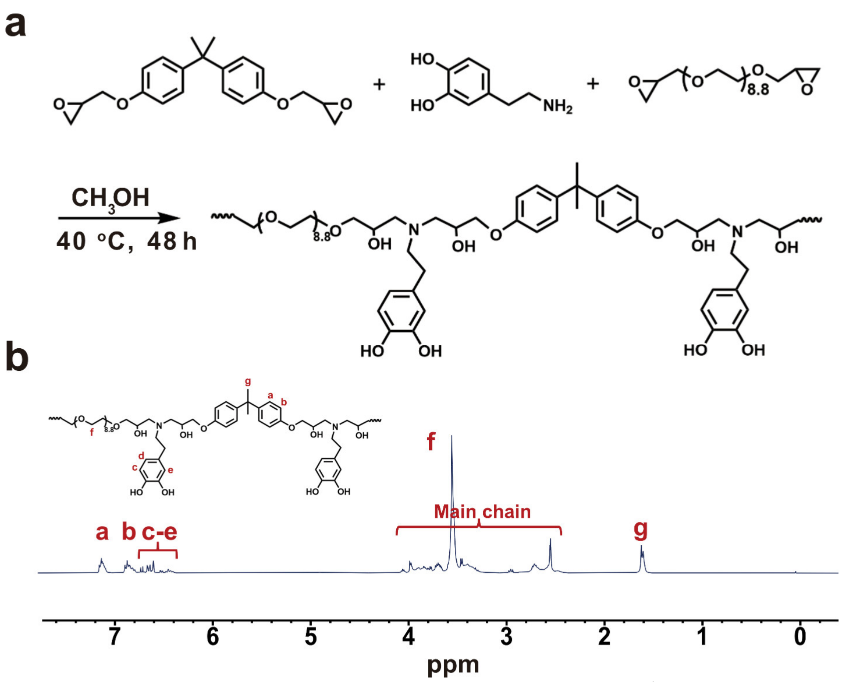

Figure S1. a) Synthesis of catechol-based polymer (CP). b) The ^{1}H-NMR spectrum of CP.

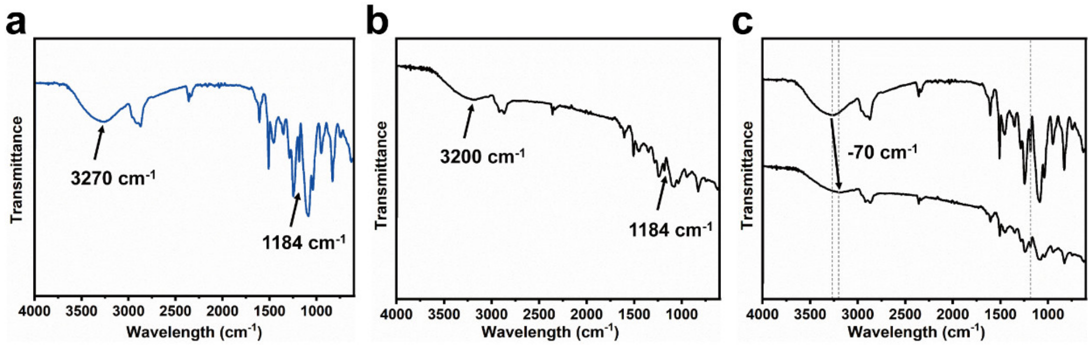

Figure S2. a) ATR FT-IR spectra of $SA_{7.5}$ and b) $SCA_{7.5}$. The Fe^{3+}-to-DOPA molar ratios were 7.5% for $SA_{7.5}$ and $SCA_{7.5}$. The AgNS content of $SCA_{7.5}$ was increased to 40 wt% for FT-IR characterization to enhance signal intensity, which was 10 wt% for the optimized formula. c) Comparison of FTIR spectra with and without AgNS.

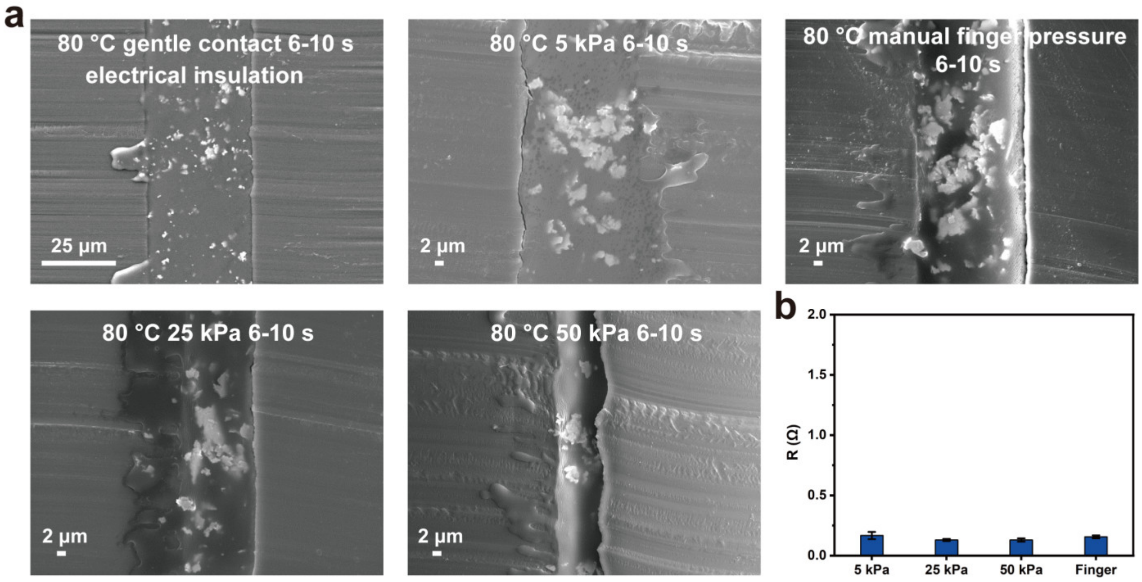

Figure S3. a) Cross-sectional SEM images of heat-pressed $SCA_{7.5}$. b) The connection resistance of SCA-connected Cu-Cu@PI under different bonding pressures.

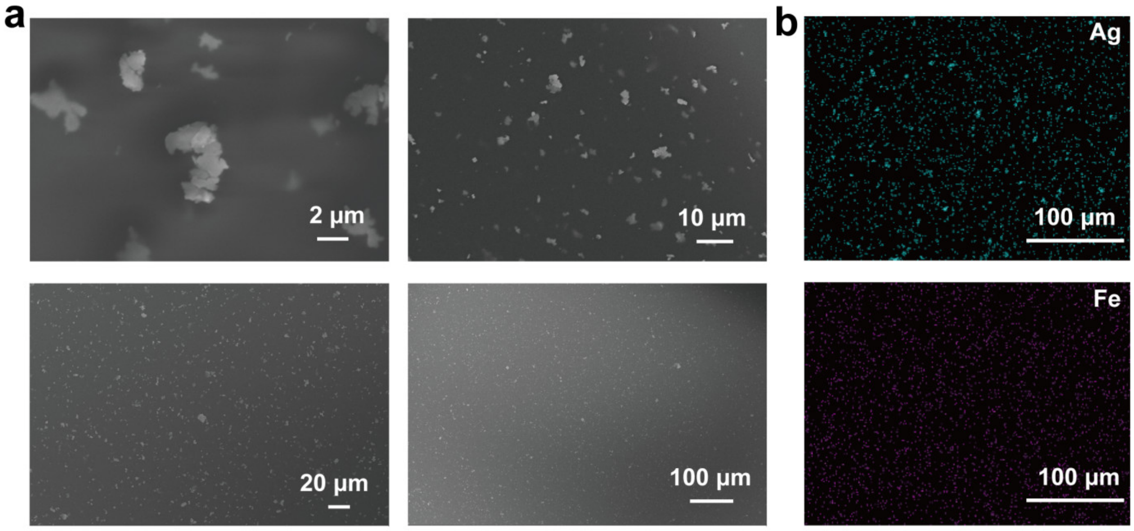

Figure S4. a) The SEM images of heat-pressed $SCA_{7.5}$. Bonding conditions: 80 °C, 6-10 s, 5 kPa. b) The EDS images of Ag and Fe in heat-pressed $SCA_{7.5}$. Bonding conditions: 80 °C, 6-10 s, 5 kPa.

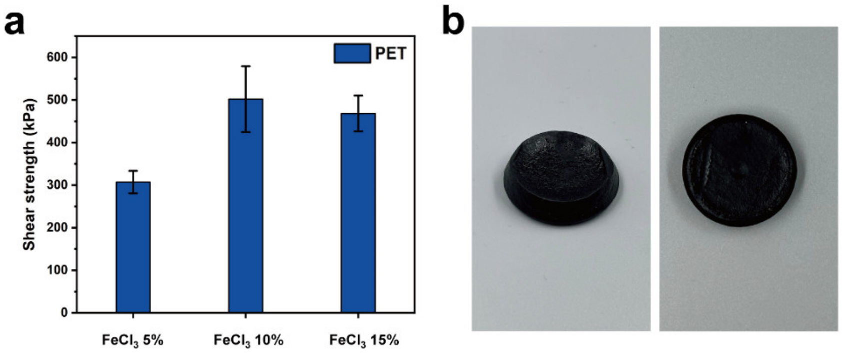

Figure S5. a) Lap shear tests of PET sheets connected by SA with different molar contents of $FeCl_3$. Bonding conditions: 80 °C, 30 min, 5 kPa. b) Photos of SA_{15} at 80 ºC.

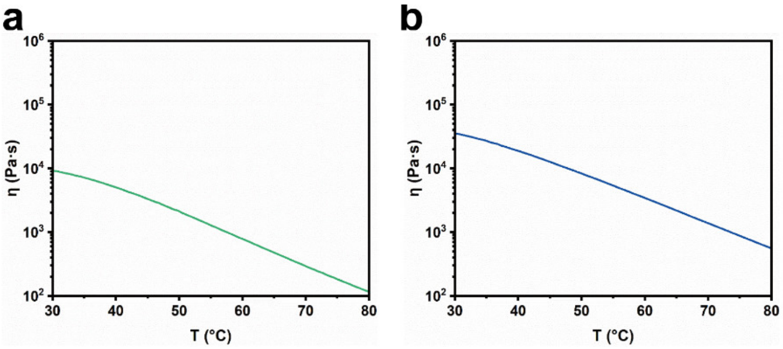

Figure S6. Viscosity of SA with a) 5% $FeCl_3$ and b) 10% $FeCl_3$ at a shear rate $\dot{\gamma} = 0.1\ s^{-1}$.

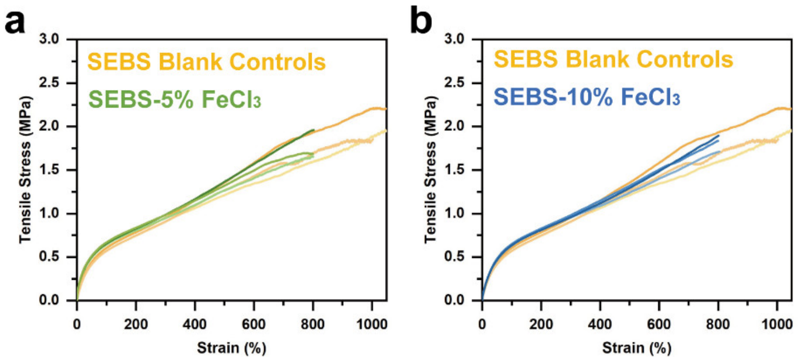

Figure S7. a) Sandwich stretching results of SA with 5% $FeCl_3$ and b) 10% $FeCl_3$. Bonding conditions: 80 °C, 6-10 s, 5 kPa.

Table S1. The channel resistance values of SA and SCA. The unit is Ω. Bonding conditions: 80 °C, 30 min, 5 kPa.

Sample \ Channel	1	2	3	4	5	6	7	8	9	Conductive channel ratio
$FeCl_3$ 5%	0.56	MΩ	MΩ	MΩ	0.22	0.42	MΩ	MΩ	0.18	44.44%
$FeCl_3$ 10%	MΩ	MΩ	2.14	MΩ	MΩ	MΩ	MΩ	0.12	0.09	33.33%
$FeCl_3$ 5% Ag 5 wt%	0.14	0.18	MΩ	0.86	0.15	MΩ	0.32	0.13	0.10	77.78%
$FeCl_3$ 5% Ag 10 wt%	0.25	0.13	0.14	0.13	0.30	0.39	0.12	0.72	0.13	100%
$FeCl_3$ 10% Ag 5 wt%	0.19	0.11	0.12	0.09	0.14	MΩ	0.12	MΩ	MΩ	66.67%
$FeCl_3$ 10% Ag 10 wt%	0.28	7.60	0.26	0.40	0.24	0.45	0.17	0.16	2.56	100%

Table S2. The resistance values of SCA over time. The unit is Ω. Bonding conditions: 80 °C, 30 min, 5 kPa.

Sample \ Number	1	2	3	4	5	6	7	8	9	Average*
$FeCl_3$ 5% Ag 10 wt% (Day1)	0.25	0.13	0.14	0.13	0.30	0.39	0.12	0.72	0.13	0.26±0.20
$FeCl_3$ 5% Ag 10 wt% (Day10)	0.18	0.14	0.16	0.14	0.16	0.20	1.05	0.14	0.14	0.26±0.30
$FeCl_3$ 7.5% Ag 10 wt% (Day1)	0.12	0.11	0.12	0.12	0.17	0.15	0.13	0.13	0.08	0.13±0.03
$FeCl_3$ 7.5% Ag 10 wt% (Day10)	0.16	0.15	0.17	0.13	0.13	0.15	0.14	0.15	0.17	0.15±0.02
$FeCl_3$ 10% Ag 10 wt% (Day1)	0.28	7.60	0.26	0.40	0.24	0.45	0.17	0.16	2.56	0.26±0.11*
$FeCl_3$ 10% Ag 10 wt% (Day2)	2.67	2.91	0.22	8.29	MΩ	MΩ	0.11	0.15	0.77	-

*Red values were not included for calculating average values for better illustration in Figure 2f.

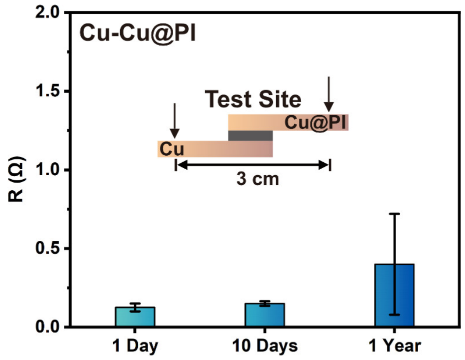

Figure S8. The connection resistance of SCA-connected Cu-Cu@PI with prolonged storage (FeCl3 7.5% Ag 10 wt%). Bonding conditions: 80 °C, 30 min, 5 kPa.

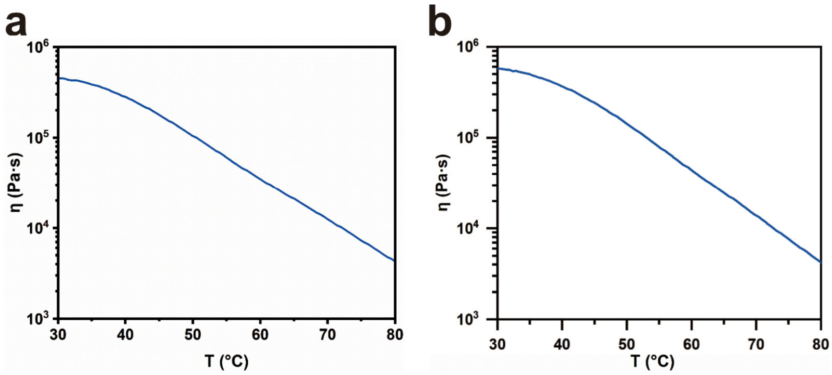

Figure S9. Viscosity of SCA with 10 wt% of AgNS and a) 5% $FeCl_3$ b) 7.5% $FeCl_3$, at a shear rate $\dot{\gamma} = 0.1\ s^{-1}$.

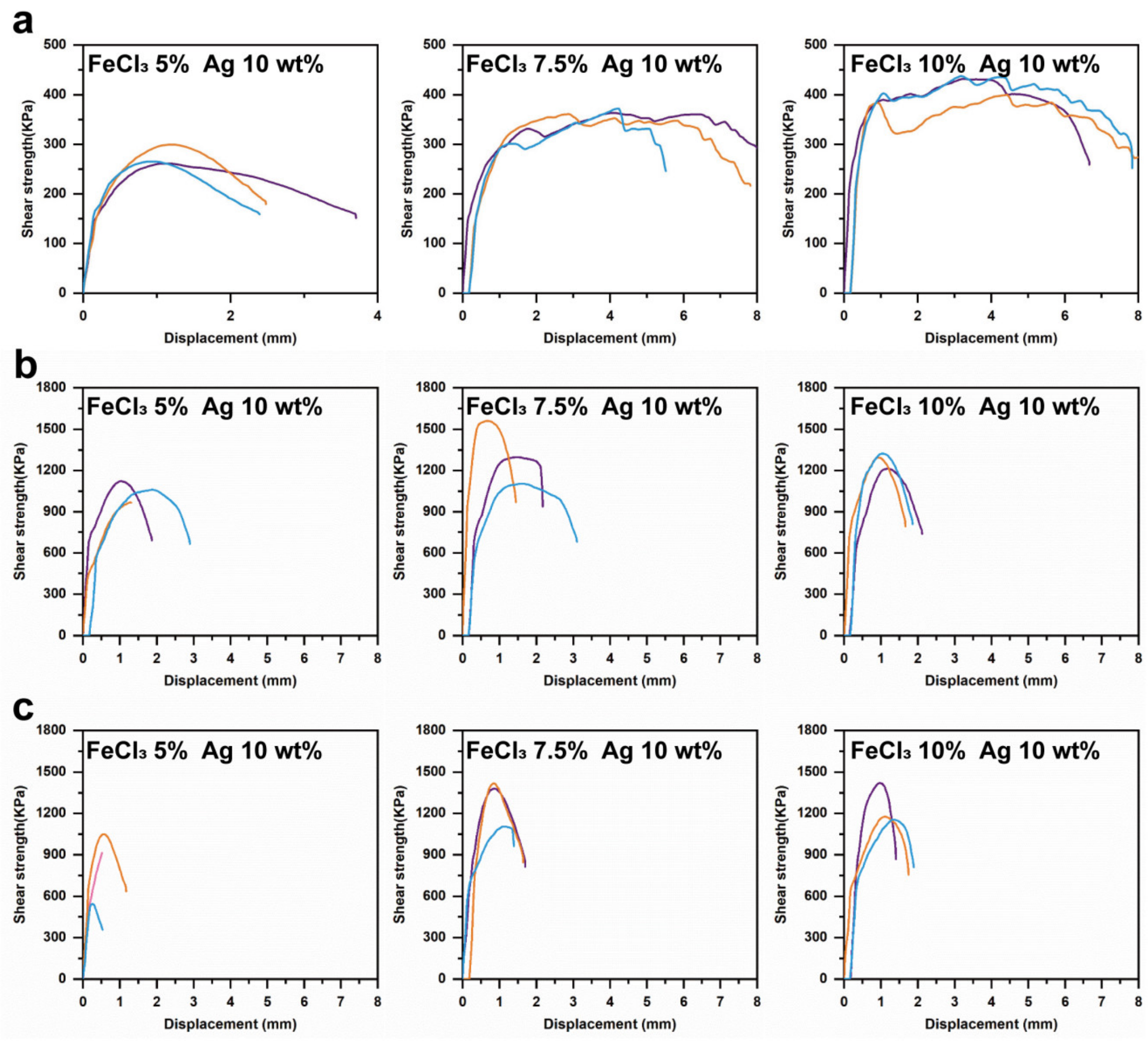

Figure S10. SCA was tested for lap shear strength on a) PET, b) Cu, and c) Ti at $FeCl_3$ contents of 5%, 7.5%, and 10%. Bonding conditions: 80 °C, 30 min, 5 kPa.

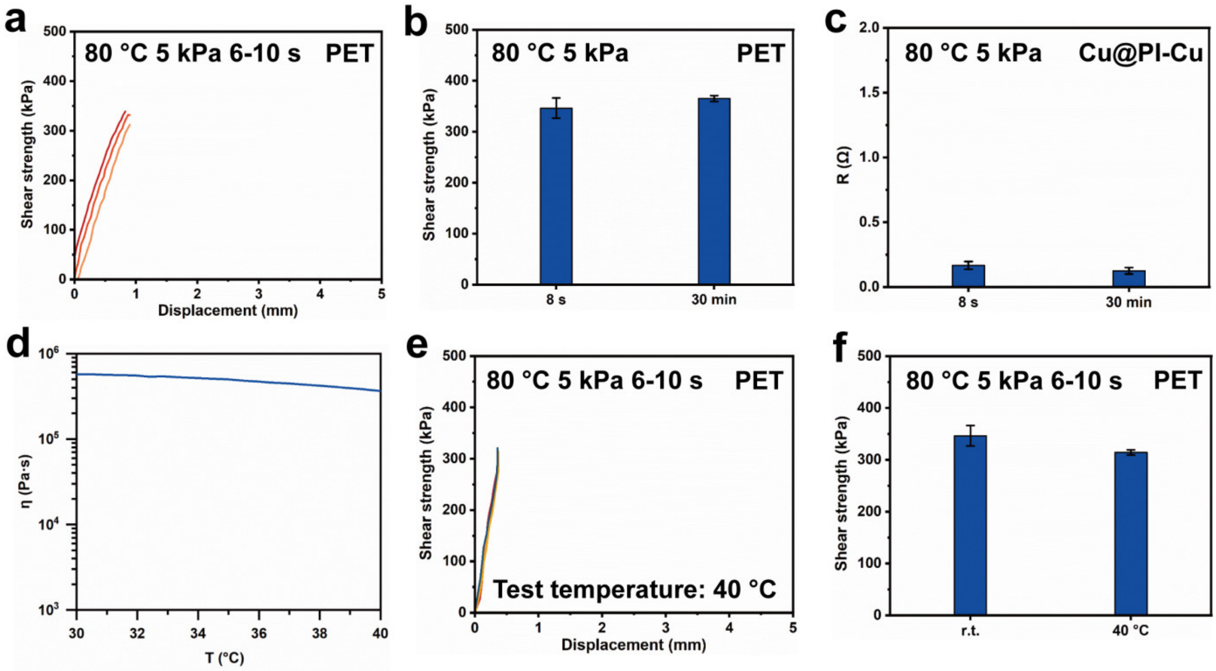

Figure S11. a) $SCA_{7.5}$ was tested for lap shear strength on PET. b) Lap shear tests of PET sheets connected by $SCA_{7.5}$ with various bonding times. c) The connection resistance of SCA-connected Cu-Cu@PI with various bonding times. d) Viscosity of $SCA_{7.5}$ at a shear rate $\dot{\gamma} = 0.1$ s^{-1} over the temperature range of 30-40 °C. e) $SCA_{7.5}$ was tested for lap shear strength on PET at a test temperature of 40 °C. f) The lap shear strength of $SCA_{7.5}$ on PET was compared at room temperature and 40 °C.

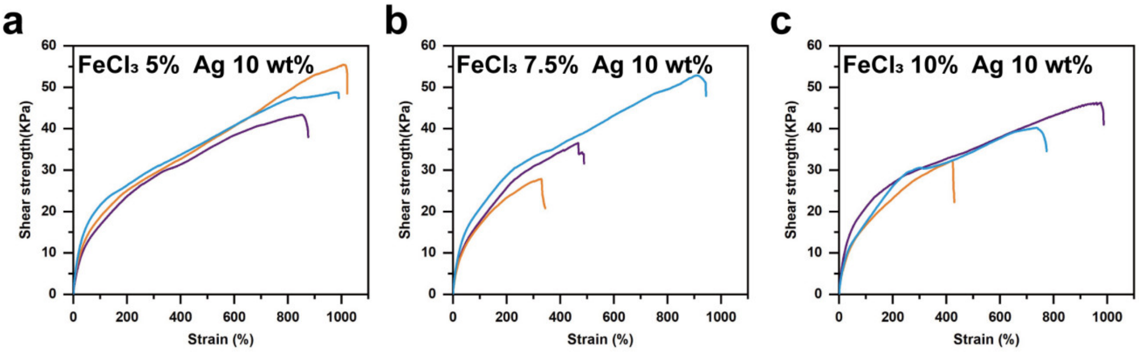

Figure S12. Lap shear tests of SEBS films connected by SCA with $FeCl_3$ contents of a) 5%, b) 7.5%, and c) 10%. Bonding conditions: 80 °C, 6-10 s, 5 kPa.

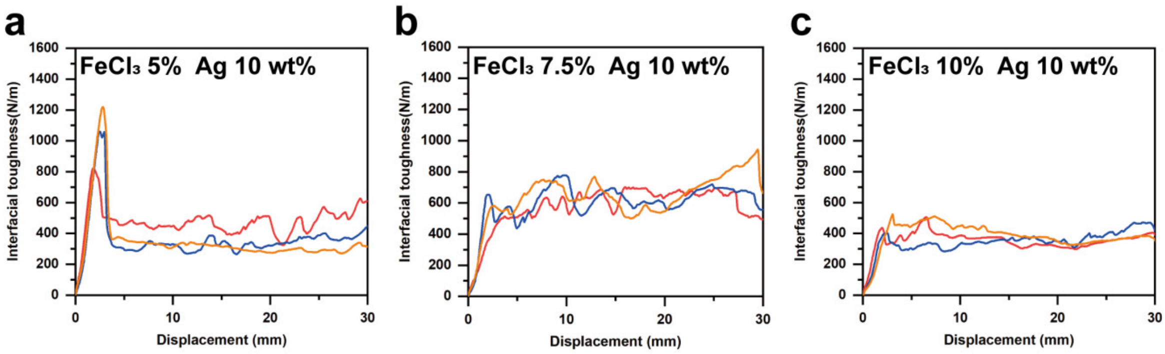

Figure S13. 180° peel tests of SEBS-SEBS connected by SCA with $FeCl_3$ contents of a) 5%, b) 7.5%, and c) 10%. Bonding conditions: 80 °C, 6-10 s, 5 kPa.

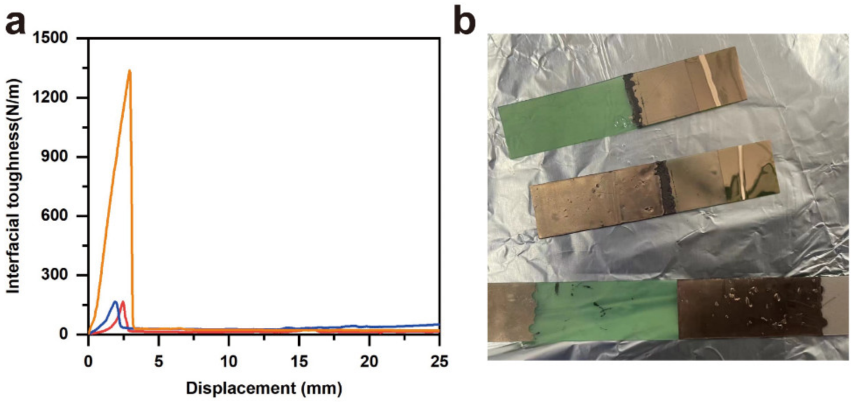

Figure S14. a) 180° peel tests of Au@SEBS-Au@SEBS connected by SCA. Bonding conditions: 80 °C, 6-10 s, 5 kPa. b) The photo after 180° peeling demonstrated that SCA remains firmly adhered to the gold layer; the interfacial failure was due to the fragile interfacial adhesion between gold and SEBS that led to complete separation of the gold layer from the stretchable SEBS substrate.

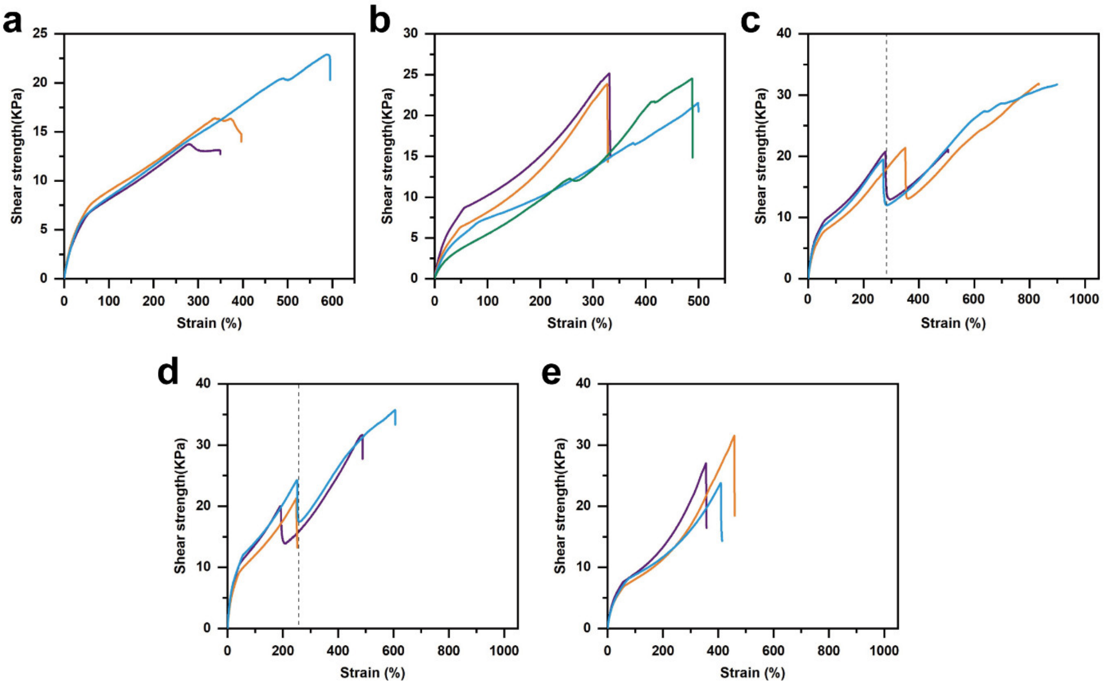

Figure S15. Lap shear tests of a) Au@SEBS-Au@SEBS, b) Au@SEBS-PI, c) Au@SEBS-PET, d) Au@SEBS-Cu, and e) Au@SEBS-Ti connected by SCA. The first breakpoints in c and d correspond to the results in Figure 3c. Bonding conditions: 80 °C, 6-10 s, 5 kPa.

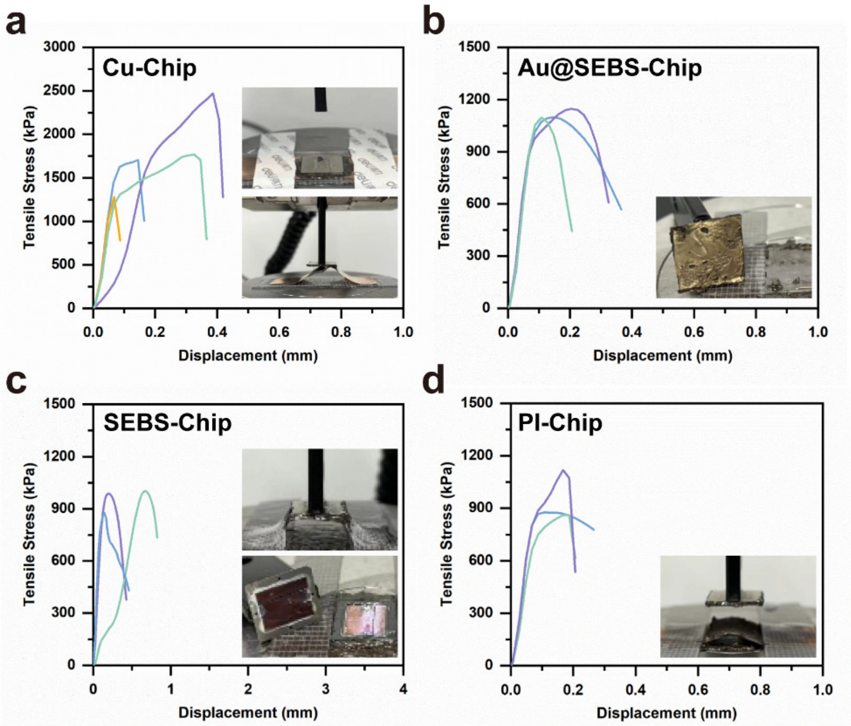

Figure S16. Adhesion strength tests of chips to common materials used in flexible circuits with SCA. Bonding conditions: 80 °C, 6-10 s, 5 kPa. a) The upper holder would break from the chip, or the Cu would detach from the lower holder before the chip detaches from Cu. b) The Au would always be peeled from the SEBS. c) The SEBS would detach from the lower holder before the chip detaches from SEBS. And in a few tests, the chips would be torn apart. d) The PI would detach from the lower holder before the chip detaches from the PI. In all these curves, the failure was not at the SCA-connected interfaces, indicating that the maximum force in each curve was below the actual adhesion force.

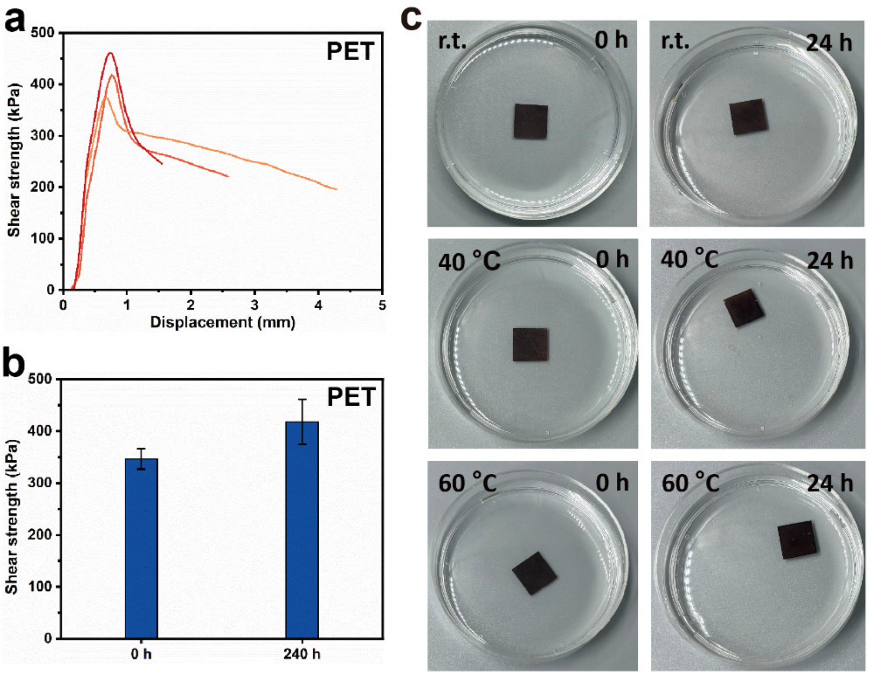

Figure S17. a) SCA was tested for lap shear strength on PET after aging at 45 °C and 85% RH for 240 h. Bonding conditions: 80 °C, 6-10 s, 5 kPa. b) Comparison of lap shear strength of SCA on PET before and after aging at 45 °C and 85% RH. Bonding conditions: 80 °C, 6-10 s, 5 kPa. c) Images of PET-SCA-PET sandwich structure samples before and after immersion in deionized water at room temperature, 40 °C, and 60 °C, respectively.

Table S3. Anisotropic conductivity tests. Bonding conditions: 80 °C, 6-10 s, 5 kPa.

N	1	2	3	4	5	6	7	8	9	10
R_1/Ω	12.5	21.6	12.0	12.9	12.3	14.0	21.0	12.3	13.6	11.8
R_2/Ω	\	\	\	\	\	\	\	\	\	\
R_3/Ω	\	\	\	\	\	\	\	\	\	\

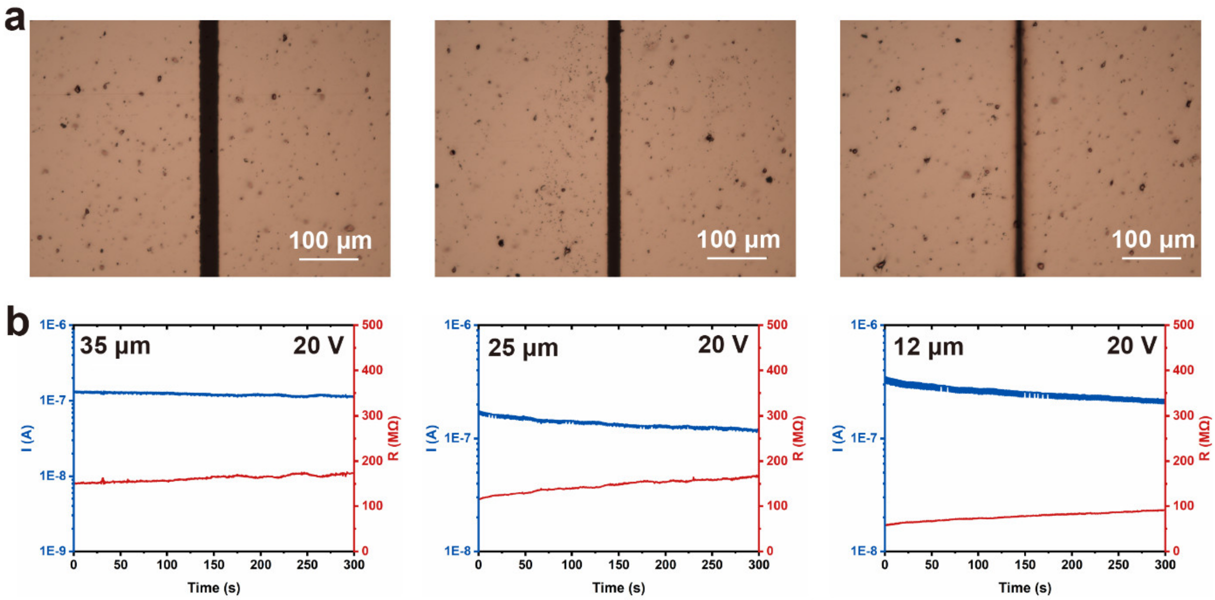

Figure S18. a) Optical microscopy image of Ag@PET electrodes with gaps of 35 μm, 25 μm, and 12 μm. b) Resistance of SCA-bonded Ag@PET-PET with gaps of 35 μm, 25 μm, and 12 μm. Bonding conditions: 80 °C, 6-10 s, 5 kPa.

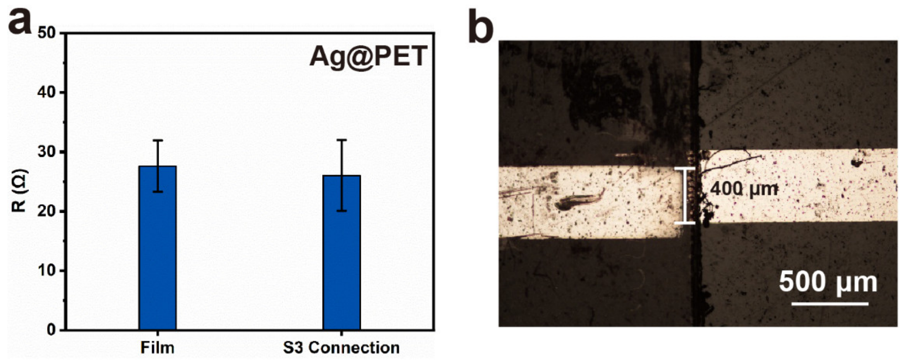

Figure S19. a) Comparison of resistance between bare Ag@PET film and SCA-connected Ag@PET. Bonding conditions: 80 °C, 6-10 s, 5 kPa. b) Optical microscopy image of Ag@PET electrodes with a contact width of 400 μm.

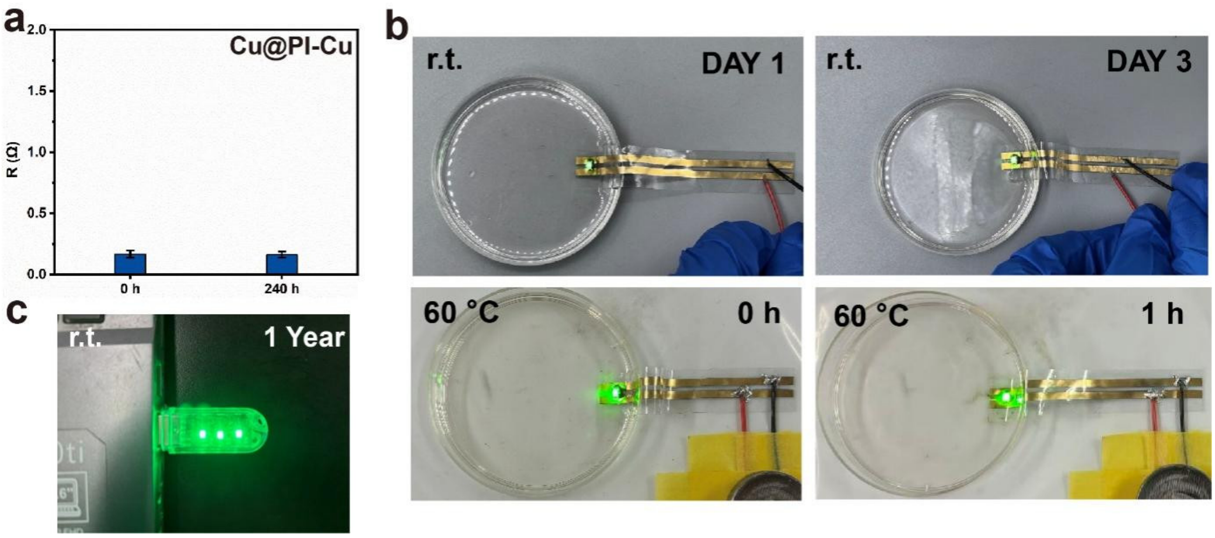

Figure S20. a) Comparison of the connection resistance of SCA-connected Cu-Cu@PI before and after aging at 45 °C and 85% RH for 240 h. Bonding conditions: 80 °C, 6-10 s, 5 kPa. b) Images of the LED-Au@SEBS flexible circuit before and after immersion in deionized water and upon heating. c) Image of the USB lamp fabricated in Figure 5b after one year of normal storage in Suzhou without additional protection measures.

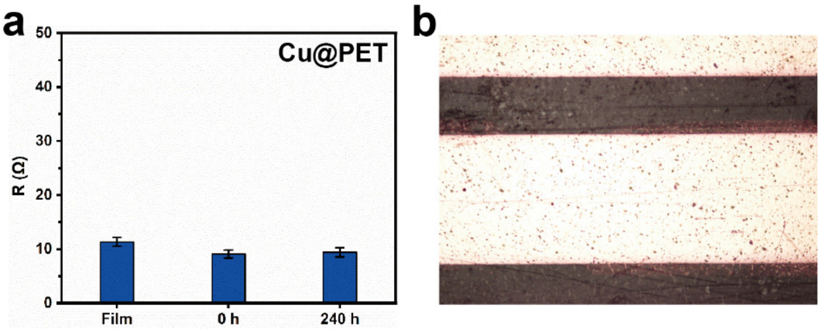

Figure S21. a) Comparison of vertical-direction connection resistance of SCA-connected Cu@PET before and after aging at 45 °C and 85% RH. Bonding conditions: 80 °C, 6-10 s, 5 kPa. b) Optical microscopy image of SCA-connected Cu@PET.

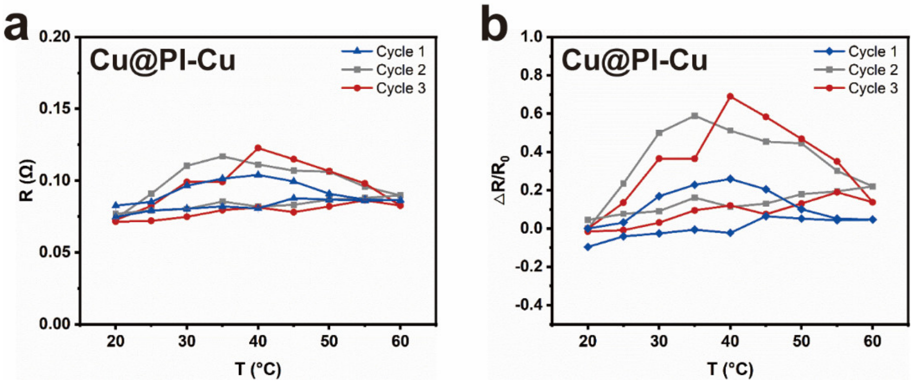

Figure S22. a) The connection resistance of SCA-connected Cu@PI-Cu under thermal cycling between 20 °C and 60 °C. Bonding conditions: 80 °C, 6-10 s, 5 kPa. b) Resistance change rate of SCA-connected Cu@PI-Cu under thermal cycling between 20 °C and 60 °C. Bonding conditions: 80 °C, 6-10 s, 5 kPa.

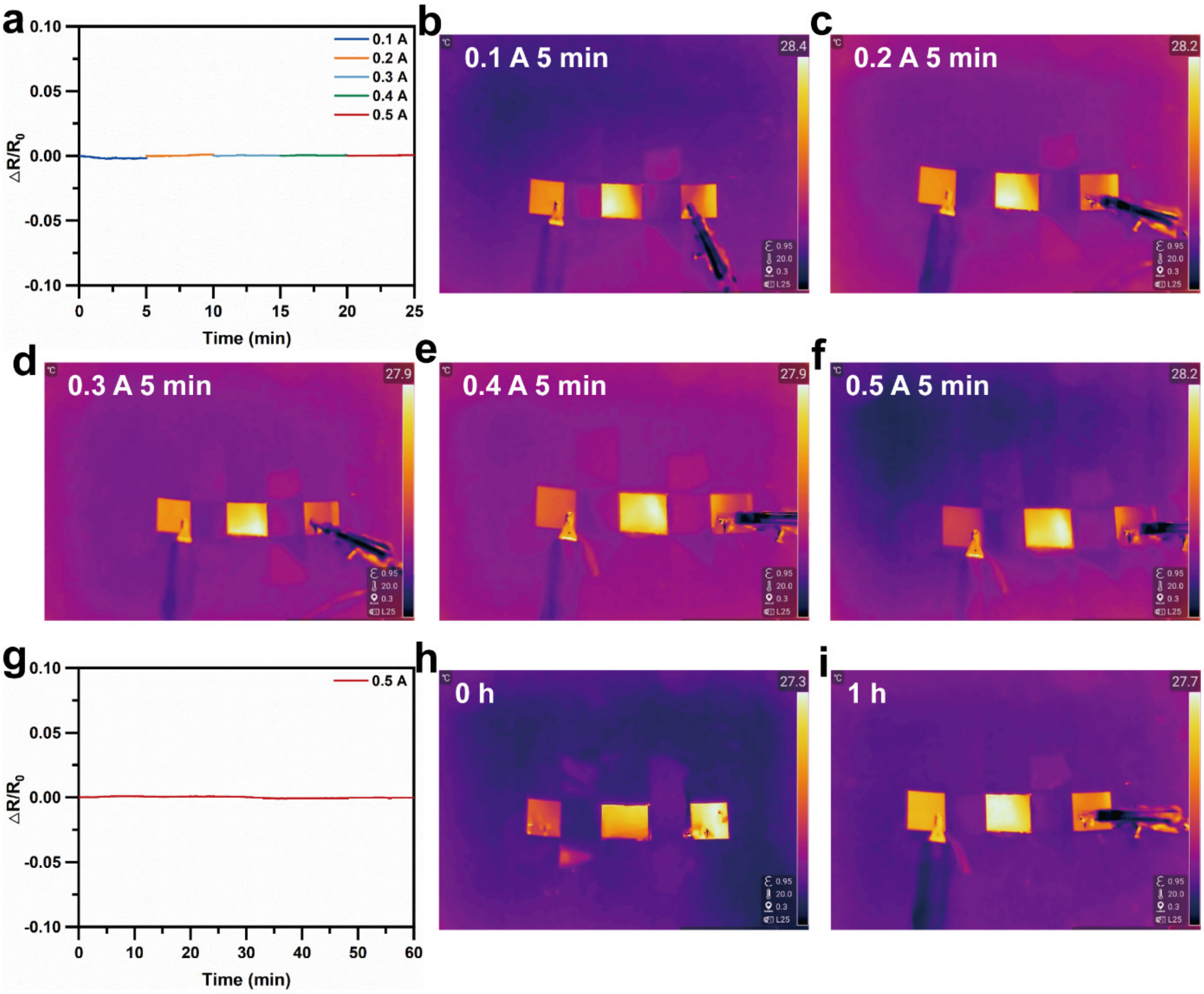

Figure S23. a) $\Delta R/R_0$ of SCA-connected Cu under current loading from 0.1 A to 0.5 A. Bonding conditions: 80 °C, 6-10 s, 5 kPa. Thermal images of SCA-connected Cu under current loading: b) 0.1 A, c) 0.2 A, d) 0.3 A, e) 0.4 A, f) 0.5 A. g) $\Delta R/R_0$ of SCA-connected Cu under a current load of 0.5 A. Bonding conditions: 80 °C, 6-10 s, 5 kPa. Thermal images of SCA-connected Cu h) before and i) after current loading at 0.5 A.

Table S4. Comparison of the conductivity of Au@SEBS films (test length: 1 cm, width: 0.6 cm) and SCA-connected Au@SEBS films (test length: 3 cm, width: 0.6 cm). Bonding conditions: 80 °C, 6-10 s, 5 kPa.

N	1	2	3	4	5	6	Average Resistance/Ω	Average Ω/cm
SEBS-Au	7.96	6.51	8.06	11.25	8.95	10.33	8.84±1.72	8.84±1.72
SEBS-Au-SCA	29.52	25.46	24.61				26.53±2.62	8.84±0.87
SEBS-Au-SCA	23.3	24.1	33.5				26.97±5.67	8.99±1.89

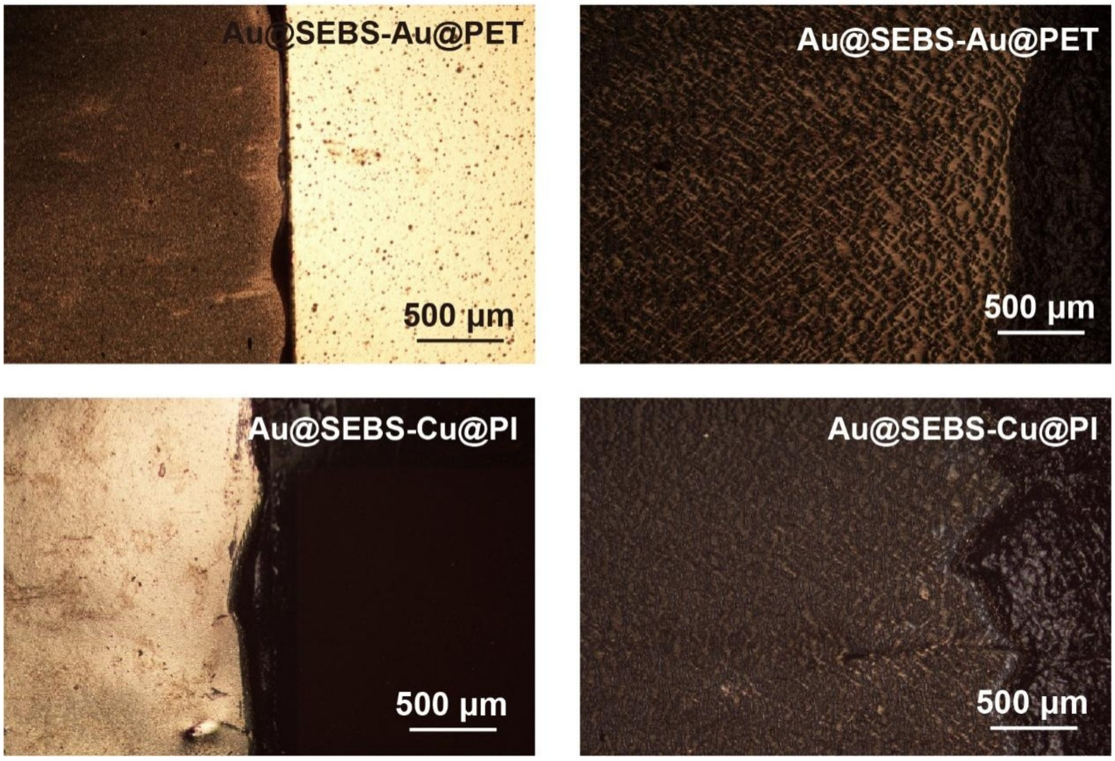

Figure S24. Optical microscopy images of the SCA-connected interface before and after stretching. Cyclic stretching inevitably leads to accumulated cracking of Au@SEBS.

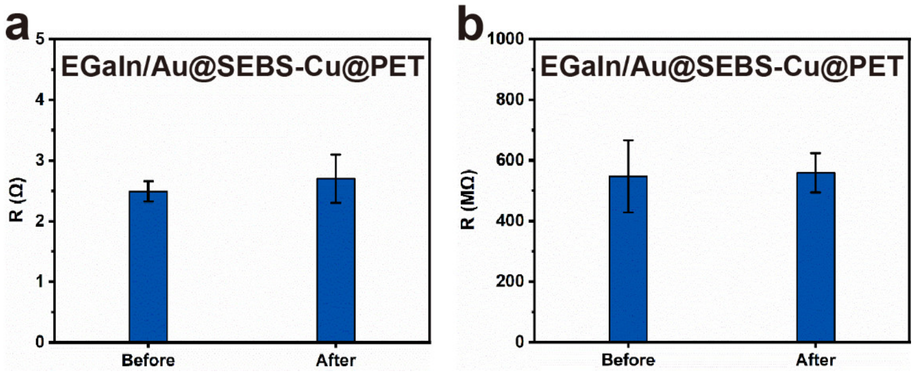

Figure S25. a) Comparison of vertical-direction connection resistance of SCA-connected EGaIn/Au@SEBS-Cu@PET before and after 10,000 cycles of stretching. Bonding conditions: 80 °C, 6-10 s, 5 kPa. b) Comparison of horizontal-direction connection resistance of SCA-connected EGaIn/Au@SEBS-Cu@PET before and after 10,000 cycles of stretching. Bonding conditions: 80 °C, 6-10 s, 5 kPa.

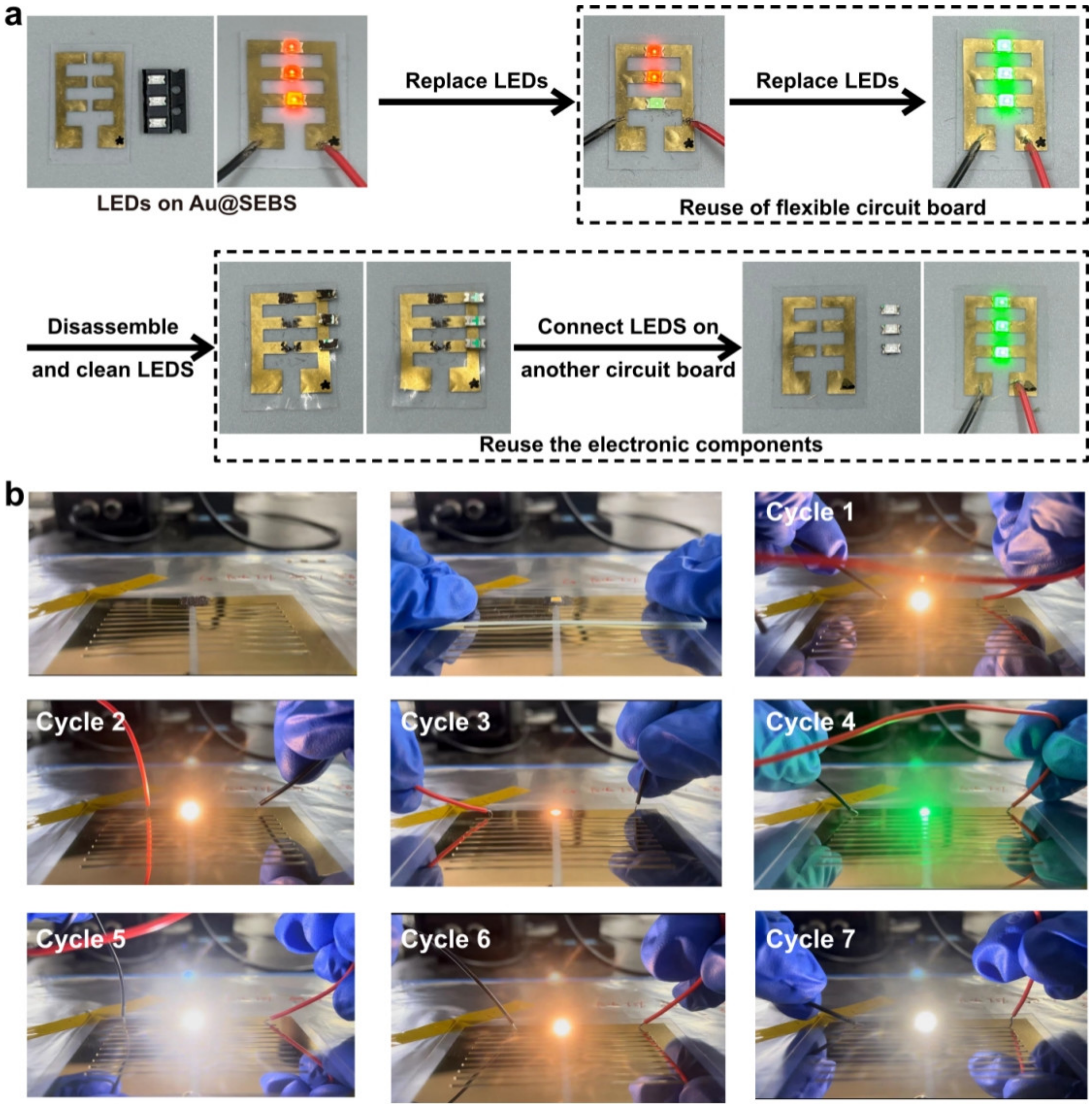

Figure S26. a) The replacement and recycling/reuse of LEDs in SCA-connected stretchable circuits. Bonding conditions: 80 °C, 6-10 s, manual finger pressure (estimated ca. 4-8 kPa). b) Different LEDs were replaced on Au@PET on the same site by SCA. The SCA was used without addition or refreshment for more than 7 cycles. The luminance of LEDs was different as they had different nominal voltages. Bonding conditions: 80 °C, 6-10 s, manual finger pressure (estimated ca. 4-8 kPa).

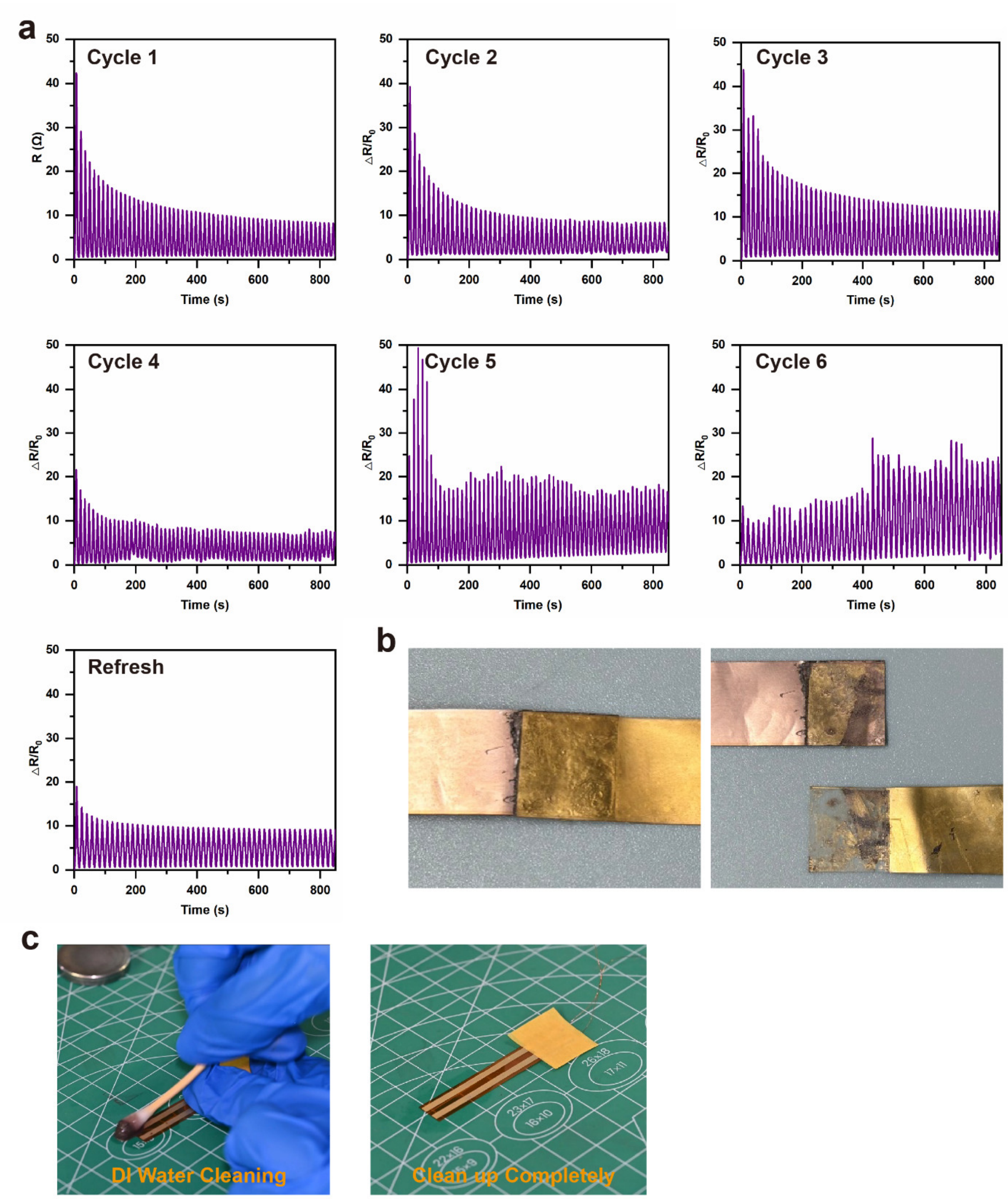

Figure S27. a) The electrical stretchability of the SCA-connected Au@SEBS-Cu@PI after each reuse without adding new adhesive. The cyclic strain was 40%. Bonding conditions: 80 °C, 6-10 s, 5 kPa. b) Images of Au peeling from SEBS due to the weak Au-SEBS interfacial adhesion upon progressive reduction of SCA. c) Complete cleaning of the test site of Cu@PI after use, which simulated the connectors of PCBs.

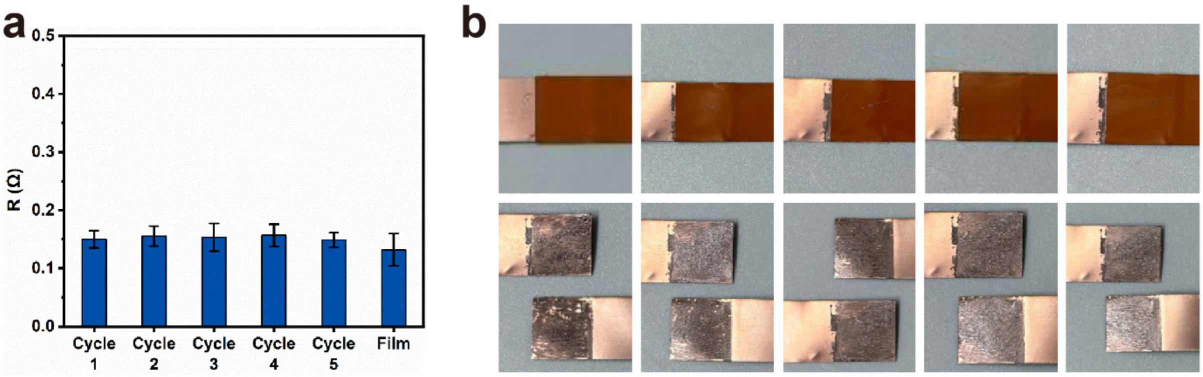

Figure S28. a) Resistance of the SCA-connected Cu@PI-Cu@PI after each reuse without adding new adhesive. Bonding conditions: 80 °C, 6-10 s, 5 kPa. b) Images of the SCA-connected Cu@PI-Cu@PI after each reuse without adding new adhesive.

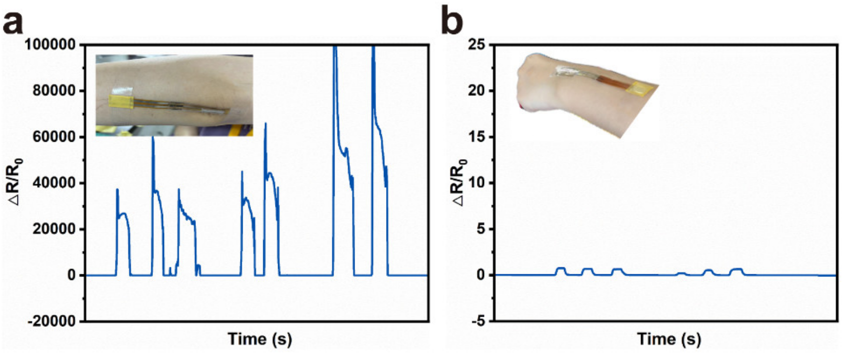

Figure S29. a) Use strain sensor 1 to test the degree of elbow bending. b) Use strain sensor 3 to test the degree of wrist bending.
